# Differential DNA methylation and changing cell-type proportions as fibrotic stage progresses in NAFLD

**DOI:** 10.1186/s13148-021-01129-y

**Published:** 2021-08-05

**Authors:** Nicholas D. Johnson, Xiumei Wu, Christopher D. Still, Xin Chu, Anthony T. Petrick, Glenn S. Gerhard, Karen N. Conneely, Johanna K. DiStefano

**Affiliations:** 1 Department of Human Genetics, Emory University, Atlanta, GA USA; 2 Population Biology, Ecology, and Evolution Program, Emory University, Atlanta, GA USA; 3 Diabetes and Fibrotic Disease Unit, Translational Genomics Research Institute, Phoenix, AZ USA; 4 Geisinger Obesity Institute, Danville, PA USA; 5 Lewis Katz School of Medicine, Temple University School of Medicine, Philadelphia, PA USA

**Keywords:** Methylation, NAFLD, NASH, Steatosis, Epigenetics, Fibrosis, Liver, Human, RNA-sequencing

## Abstract

**Background:**

Non-alcoholic fatty liver disease (NAFLD) is characterized by changes in cell composition that occur throughout disease pathogenesis, which includes the development of fibrosis in a subset of patients. DNA methylation (DNAm) is a plausible mechanism underlying these shifts, considering that DNAm profiles differ across tissues and cell types, and DNAm may play a role in cell-type differentiation. Previous work investigating the relationship between DNAm and fibrosis in NAFLD has been limited by sample size and the number of CpG sites interrogated.

**Results:**

Here, we performed an epigenome-wide analysis using Infinium MethylationEPIC array data from 325 individuals with NAFLD, including 119 with severe fibrosis and 206 with no histological evidence of fibrosis. After adjustment for latent confounders, we identified 7 CpG sites whose DNAm associated with fibrosis (*p* < 5.96 × 10^–8^). Analysis of RNA-seq data collected from a subset of individuals (*N* = 56) revealed that gene expression at 288 genes associated with DNAm at one or more of the 7 fibrosis-related CpGs. DNAm-based estimates of cell-type proportions showed that estimated proportions of natural killer cells increased, while epithelial cell proportions decreased with disease stage. Finally, we used an elastic net regression model to assess DNAm as a biomarker of fibrotic stage and found that our model predicted fibrosis with a sensitivity of 0.93 and provided information beyond a model based solely on cell-type proportions.

**Conclusion:**

These findings are consistent with DNAm as a mechanism underpinning or marking fibrosis-related shifts in cell composition and demonstrate the potential of DNAm as a possible biomarker of NAFLD fibrosis.

**Supplementary Information:**

The online version contains supplementary material available at 10.1186/s13148-021-01129-y.

## Background

Non-alcoholic fatty liver disease (NAFLD), which encompasses a group of conditions characterized by the accumulation of fat in liver cells, is the most common chronic liver disease in Western countries [1]. Hallmarks of NAFLD include excessive lipid storage in hepatocytes and persistent wound healing carried out by activated myofibroblasts [2]. While the underlying causes are not fully known, risk factors for NAFLD include obesity and insulin resistance [3]. A subset of NAFLD patients also develop inflammation and fibrosis, collectively representing non-alcoholic steatohepatitis (NASH), an advanced form of NAFLD that is associated with increased liver-related morbidity and mortality [4]. NASH patients have a greater risk of developing cirrhosis and hepatocellular carcinoma [5] by processes involving interactions of many hepatic cell types, including parenchymal and non-parenchymal cells. Few studies have focused on epigenetic changes in advanced fibrosis in NAFLD [6].

DNA methylation (DNAm) is an epigenetic DNA modification in which a cytosine residue predominantly followed by a guanine, i.e., a CpG dinucleotide, is modified through the covalent addition of a methyl group. DNAm has been shown to play a regulatory role in fibrogenesis in a variety of organs [7], but its contribution to liver fibrosis is not well characterized [8]. In cell culture, transdifferentiation of hepatic stellate cells into myofibroblasts, the primary cellular mediators of liver fibrosis, is accompanied by changes in DNAm status of hundreds of genes [9]. Previous studies have interrogated NAFLD-related DNAm status of specific candidate genes [10, 11]. For example, DNAm of the peroxisome proliferator-activated receptor γ (PPARγ) gene promoter has been found to associate with severe versus mild fibrosis in NAFLD [11]. Extending beyond candidate genes, Gerhard et al. [12] performed an epigenome-wide association study (EWAS) to compare DNAm between advanced fibrosis (*N* = 14) versus sex- and age-matched non-fibrotic liver samples (*N* = 15) and observed differential DNAm at 208 CpG islands, as well as 34 CGI-transcript pairs showing significant association between DNAm and gene expression. This study also found differential DNAm to be enriched for biological pathways relevant to cirrhosis. Another EWAS of liver samples (35 healthy, 34 with simple steatosis, and 26 with NASH) reported 1292 CpG sites with NASH-associated differential DNAm [13].

To date, studies of NAFLD and DNAm in liver have been limited to small samples, while larger EWAS of NAFLD phenotypes has been performed in less invasive tissues such as blood. In a blood EWAS interrogating the association between DNAm and hepatic fat accumulation, Ma et al. [14] observed 58 CpGs with significant associations (FDR < 0.05) in a discovery cohort (*N* = 1496), among which 22 were significant in a replication cohort (*N* = 1904). A Mendelian randomization analysis from this study suggested that one of the CpGs may be causally related to NAFLD. Other studies have reported results showing NAFLD-associated changes in DNAm, although all of these have been based on relatively limited sample sizes of less than 100 individuals [13, 15–18].

In addition to serving as a common and relatively stable molecular modification that functions to regulate gene expression during differentiation of cells, DNAm patterns can be used to identify distinct cell types and changes in cell-type composition [19], a well-known feature of NAFLD disease progression [20–22]. Because DNAm is usually tissue- and cell-type-specific, cellular composition is a major contributor to DNAm patterns. Changes in cellular composition can thus be inferred from changes in DNAm profiles, through deconvolution of cell-type-specific loci. Progression to fibrosis in NAFLD may involve changes in a variety of cell types, including hepatocytes, Kupffer cells, stellate cells, sinusoidal endothelial cells, cholangiocytes, and various immune cells. There have been few studies that have used DNAm data to define cellular composition in the liver, and of those, most are based on mouse models [23].

Previous studies of DNAm in NAFLD have been mostly based on small sample sizes or blood-derived DNA sources [12, 13, 15–18]. Here, we sought to extend the available data by focusing specifically on the fibrotic stage in a large set of liver samples from individuals with NASH, and interrogating nearly twice as many CpG sites as previous studies. In addition, we used DNAm data to examine estimated cell-type proportions, investigated the potential of DNAm to serve as a marker of fibrotic stage, and conducted a combined analysis of DNAm and gene expression to identify possible regulatory mechanisms involved in the progression of NAFLD fibrosis.

## Results

### Patient characteristics

Demographic and clinical characteristics of the study participants are shown in Table 1. The majority (77%) of study participants were female and of European ancestry (99%) with a mean (± SD) age of 48.6 ± 11.5 and BMI of 47.1 ± 9.1. The distribution of fibrosis was 68% no fibrosis (Grade 0), 17% bridging (Grade 3), 11% incomplete cirrhosis (Grade 3/4), and 9% cirrhosis (Grade 4).

**Table 1:** Study cohort demographic information and clinical characteristics (*N* = 325)

	Fibrosis stage				
	Grade 0	Grade 3	Grade 3/4	Grade 4	All
Age (years)	47.2 (12.4)	49.5 (10.0)	50.4 (8.1)	53.8 (9.5)	48.6 (11.5)
BMI	46.6 (8.8)	48.6 (8.0)	46.5 (9.7)	48.1 (12.1)	47.1 (9.1)
Sex					
Female	166	34	29	22	251
Male	40	21	7	6	74
Race					
Black or African American	3	0	0	0	3
White	203	54	36	28	321
Not available	0	1	0	0	1
Type 2 diabetes					
No	130	7	4	4	145
Yes	76	48	32	24	180

### DNA methylation data are concordant across technical replicates and cluster by sex and disease status

PCA was used to identify and remove ten outliers (Additional file 1: Fig S1–S2). One sample had eight replicates, and nine samples had pairs of duplicates, which also clustered by individual, indicating high concordance between replicates (Additional file 1: Fig S3). Following removal of outliers and averaging of the duplicates/replicates, the final sample size was *N* = 325. PCA of the DNAm data for each sample showed that samples clustered by sex and fibrosis stage (Additional file 1: Fig S1A–C). While the first two principal components partially separated individuals by sex, male and female individuals were fully separated into distinct clusters when the 3rd principal component was added to the plot (Additional file 1: Fig S1B). Partial overlap was seen between groups of samples with fibrotic versus non-fibrotic liver tissue (Additional file 1: Fig S1C). In addition, we observed significant associations between age and PC2–PC6, along with PC9 (Additional file 1: Fig S2).

### Fibrosis has a distinct DNA methylation profile

**Fig. 1:**
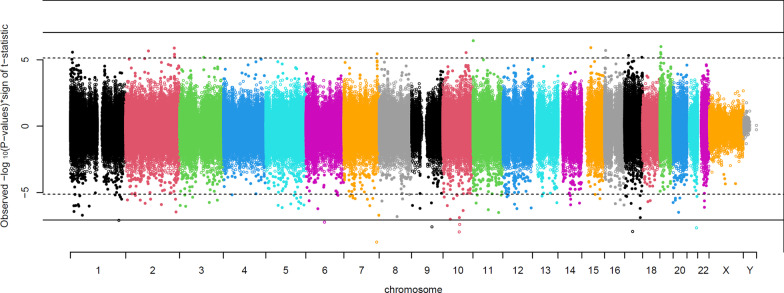
Manhattan plot depicting association between DNAm and fibrosis. The solid horizontal lines indicate the Bonferroni threshold, and the dotted lines indicate the FDR < 0.05 threshold. Covariates include age, sex, BMI, and two latent variables

**Fig. 2:**
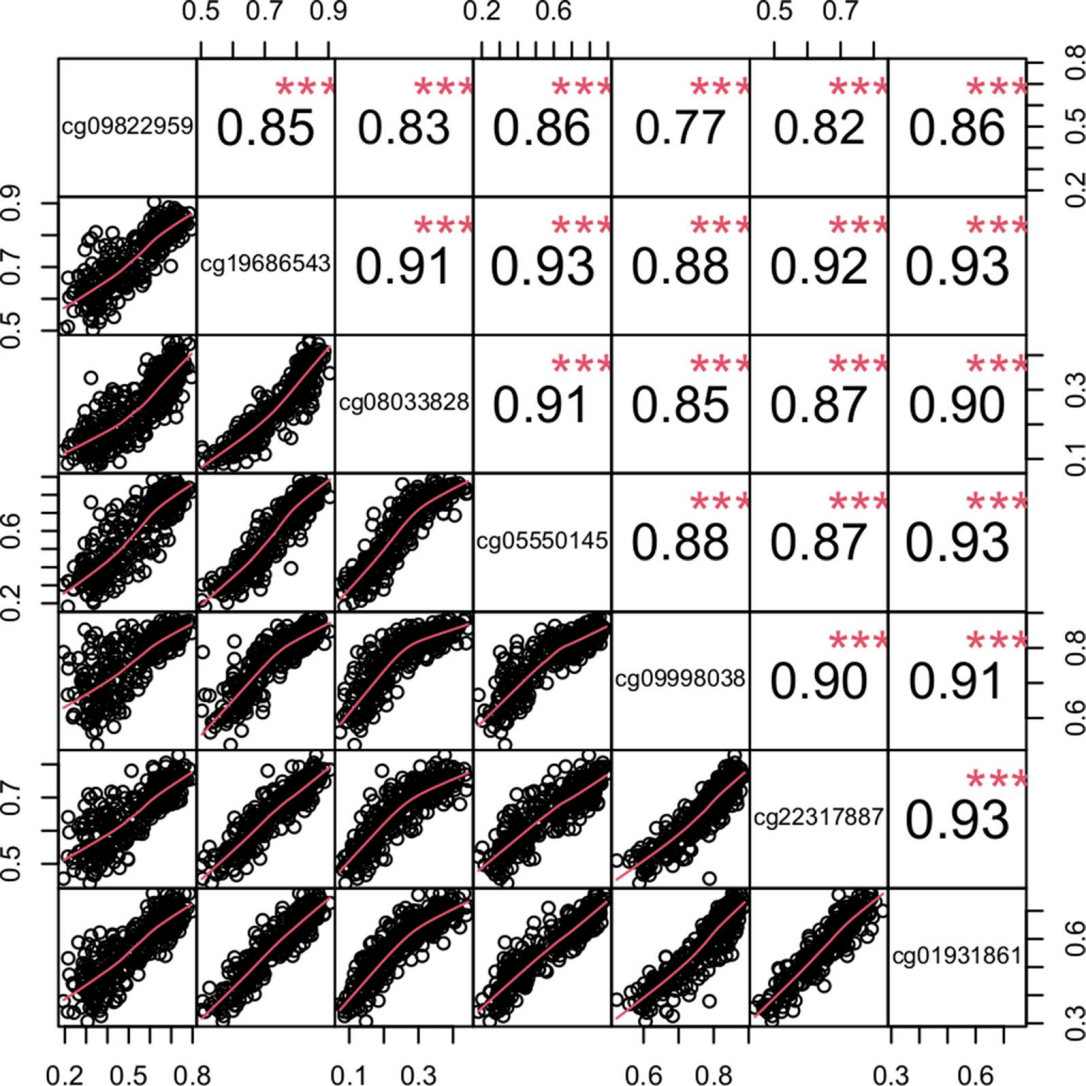
Boxplot of DNAm proportions against NAFLD fibrosis stage for the most significant CpG site (cg19686543) in the primary analysis

**Fig. 3:**
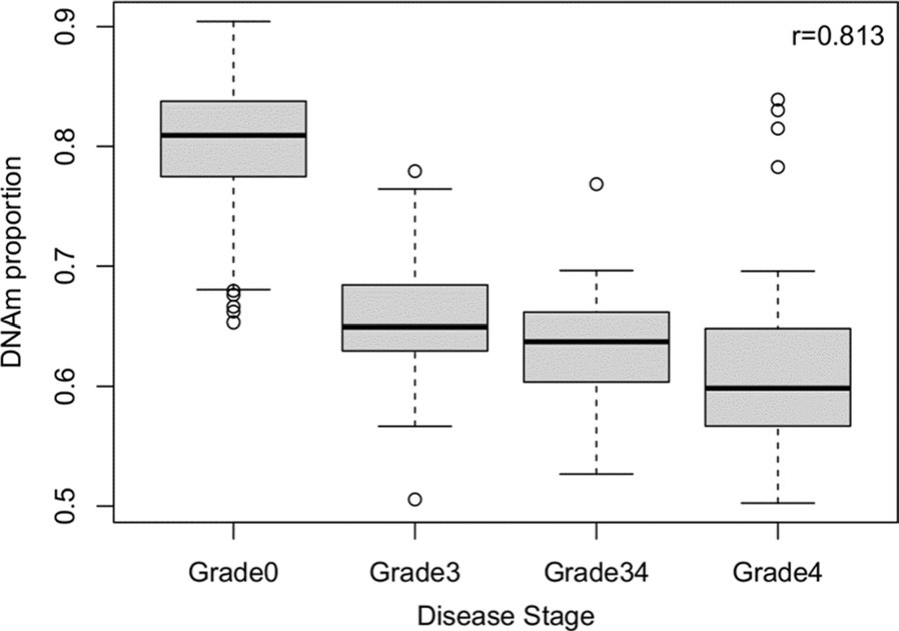
Correlation matrix of the seven NAFLD-related CpG sites from the main analysis

We performed an EWAS adjusted for age, sex, and BMI, as well as two latent confounders (see “Methods” section) using DNAm β-values as the dependent variable and the presence of fibrosis at any stage as the independent variable. Seven CpG sites passed the Bonferroni significance threshold (*p* < 5.96 × 10^–8^); all seven sites were hypomethylated in individuals with fibrosis (Fig. 1; Table 2). Although located on six different chromosomes, the seven fibrosis-related CpGs were highly correlated with one another (Fig. 2) and showed decreased DNAm levels that corresponded to increasing severity of fibrosis (top CpG sites shown in Fig. 3). Three and four CpG sites were located in genes and enhancers, respectively (Table 2). The effect sizes for these CpG sites indicated that the estimated DNAm proportion is, on average, 0.05–0.16 lower in individuals with fibrosis compared to those without.

**Table 2:** Summary information for seven CpGs associated with fibrosis

CpG	Chr	Position	Effect size	T-stat	*p* val	Overlapping gene	Enhancer ID	Genes linked to enhancer
cg09822959	6	82,862,789	− 0.159	− 5.4	5.93E−08		GH06F082862	LOC105377877, GC06M082916
cg19686543	7	148,327,678	− 0.093	− 6.0	1.87E−09	CNTNAP2	None	N/A
cg08033828	9	89,137,984	− 0.093	− 5.6	2.61E−08	SHC3	GH09F089136	SHC3, CKS2
cg05550145	10	71,871,455	− 0.174	− 5.7	1.08E−08		GH10F071871	VSIR, PSAP
cg09998038	10	73,894,805	− 0.052	− 5.5	3.99E−08		GH10F073886	NDST2, KAT6B, C10orf55, DNAJC9, FUT11, VCL, MYOZ1, CAMK2G, SEC24C, DUSP8P5, ANXA7, PLAU
cg22317887	17	37,698,375	− 0.067	− 5.7	1.17E−08	HNF1B	GH17F037697	HNF1B, MRPL45, LOC105371754, GC17P037657
cg01931861	21	41,601,636	− 0.077	− 5.6	2.22E−08		GH21F041601	LOC105372809, LOC105372812

When applying a less stringent statistical threshold, a Benjamini–Hochberg FDR cutoff of 0.05, we observed 18 hypermethylated and 110 hypomethylated CpG sites (Additional file 1: Table S1). Gene ontology (GO) analysis of these 128 CpG sites did not yield evidence of significant enrichment, although the top GO terms correspond to biological processes involved in NAFLD, including apoptosis and morphogenesis (Table 3).

**Table 3:** The top ten GO terms showing enrichment in the primary analysis

Ontology	Term	*p*
MF	ATPase-coupled heme transmembrane transporter activity	9.93E−04
BP	Heme transmembrane transport	9.93E−04
BP	Regulation of apoptotic process involved in morphogenesis	2.69E−03
BP	Regulation of apoptotic process involved in development	2.73E−03
BP	Positive regulation of miRNA catabolic process	3.64E−03
BP	Regulation of miRNA catabolic process	4.62E−03
BP	Positive regulation of stem cell differentiation	5.03E−03
BP	Regulation of steroid biosynthetic process	5.32E−03
CC	Phosphatidylinositol 3-kinase complex	5.55E−03
BP	Negative regulation of phospholipase A2 activity	5.85E−03

*MF* molecular function, *BP* biological process, *CC* cellular component

### Estimated cell-type proportions vary with disease stage

**Fig. 4:**
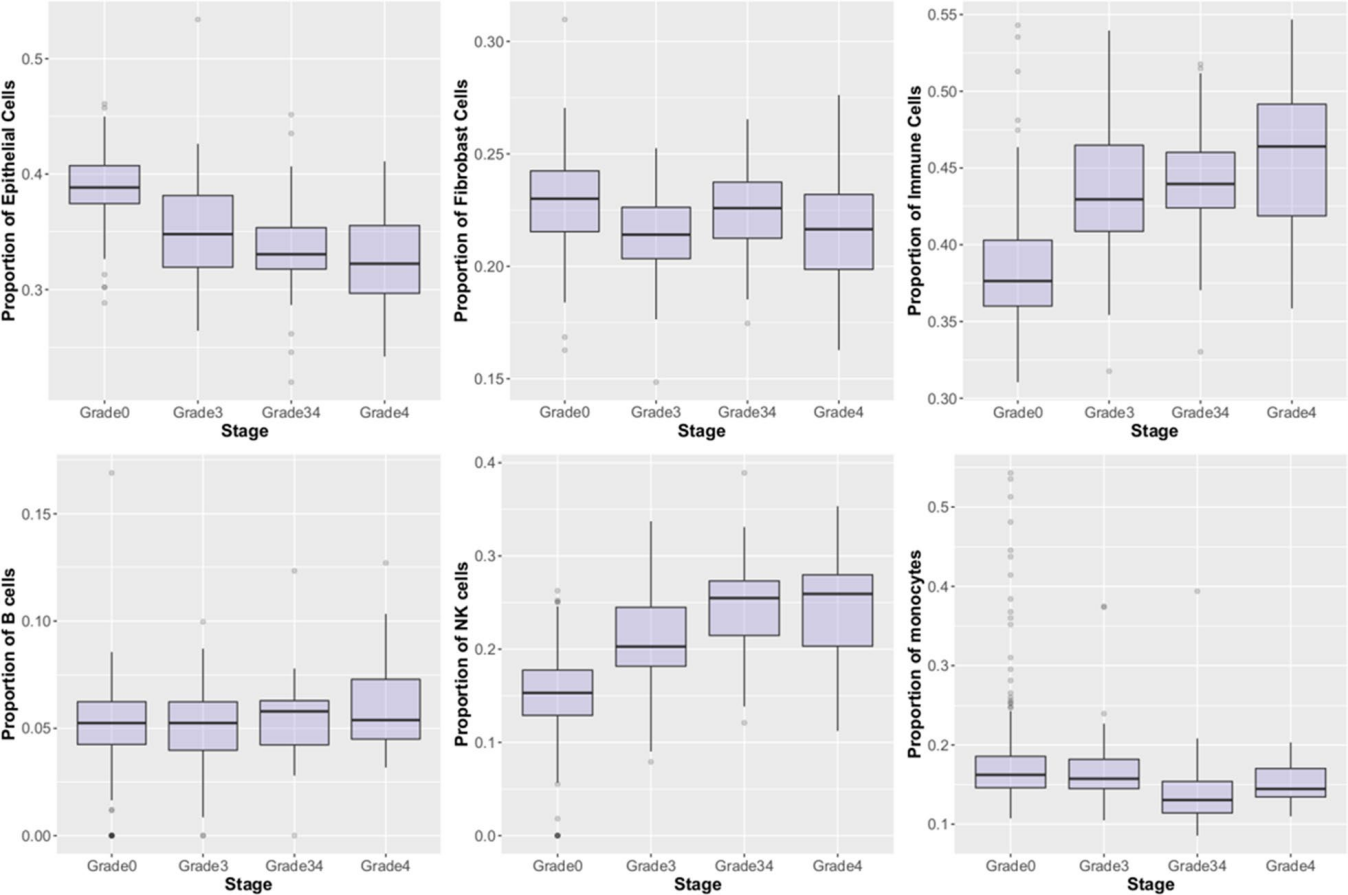
First row of panels shows estimated proportions of epithelial, fibroblast, and immune cells by fibrotic stage. Second row of panels shows estimated proportions of subsets of immune cells by fibrotic stage

Measurement of DNAm allows for the deconvolution of specific cell types from data derived from bulk tissue. We therefore sought to estimate proportions of cell types found in biopsied liver tissue using EpiDISH [24], as described in the Methods section. Our results indicated that estimated cell-type proportions change with the presence and severity of fibrosis (Fig. 4). Estimated proportions of epithelial cells decreased from approximately 38% in non-fibrotic liver to ~ 32% in cirrhosis. In contrast, the estimated proportions of immune cells increased as fibrosis stage advanced from about 40% in non-fibrotic liver to 48% in cirrhosis. We used a secondary non-overlapping EpiDISH reference set to estimate subsets of immune cells and observed that natural killer (NK) cells increased from about 16% of immune cells in non-fibrotic liver to over 25% in incomplete cirrhosis and cirrhosis.

### DNAm profile of fibrosis may partially reflect changing cell-type proportions

For comparison to our primary analysis, which adjusted for latent confounders such as cell type via a “reference-free” approach, we performed a secondary EWAS that directly adjusted for cell-type proportions estimated via a reference-based method [24] (Additional file 1: Table S2; Fig S4). The purpose of this secondary analysis was to facilitate comparisons to previous EWAS that have used similar models, and also to provide insight into the extent to which cellular composition may contribute to the latent confounding. Using this approach, we observed 25,170 hypermethylated CpG sites and 32,118 hypomethylated CpG sites that achieved statistical significance at a Bonferroni threshold of 5.96 × 10^–8^ (Additional file 1: Fig S4). A similar model unadjusted for cell-type proportions yielded an even larger set of significant CpG sites. In our primary analysis, we found no genomic inflation (*λ* = 1.0), i.e., the deviation of the distribution of the observed results compared to the distribution of the expected results. In the secondary analysis (Additional file 1: Fig S5), results were highly inflated (*λ* = 4.28). The inflation observed when using the reference-based approach suggests that there may be significant changes in DNAm due to unmeasured confounders, such as technical factors or specific cell subtypes not measured by current reference-based methods.

Pathway analysis of the reference-based results from this secondary analysis indicated that 404 GO terms were significantly enriched according to a Bonferroni cutoff *p* < 2.2 × 10^–6^; the top ten results are shown in Additional file 1: Table S2. Some of the significant GO terms were suggestive of involvement in lipid metabolism, morphogenesis, and cell migration, which are all processes observed in NAFLD.

### Subgroup-based sensitivity analyses are largely consistent with one another

To investigate the robustness of our results, we compared the results of our primary analysis (Fig. 1) to five additional subgroup analyses (Additional file 1: Fig S6). When we re-ran the analysis including only individuals without type 2 diabetes (*N* = 145), 43 CpG sites passed the Bonferroni significance threshold, but test statistics were correlated with test statistics obtained in the original analyses (*r* = 0.608) (Additional file 1: Fig S6B). We also performed an analysis in which only individuals with Grade 0 fibrosis and Grade 4 fibrosis were included (*N* = 234), and again, the results were similar (*r* = 0.784) to five CpG sites showing differential DNAm (Additional file 1: Fig S6A). In sex-specific analyses, results were similar for men and women (*r* = 0.35), with five differentially methylated CpG sites in men and eight differentially methylated sites in women (Additional file 1: Fig S6D). We also tested for an interaction between sex and fibrosis state and observed 154 significant CpG sites (Additional file 1: Fig S6C). The effect of fibrosis on DNAm was higher in women for 49 CpGs and higher in men for 105 CpGs. None of the 154 CpGs overlapped the seven significant sites in the primary analysis. These results show that our primary analysis is robust to diabetes status and the inclusion/exclusion of particular disease stages. We did not detect sex-specific effects of fibrosis status on DNAm for the seven CpGs associated with fibrosis in the primary analysis, possibly due to the low number of males in the cohort.

### DNA methylation profile of fibrosis similar to previously published study on epigenetics of non-alcoholic steatohepatitis (NASH) in liver tissue

**Fig. 5:**
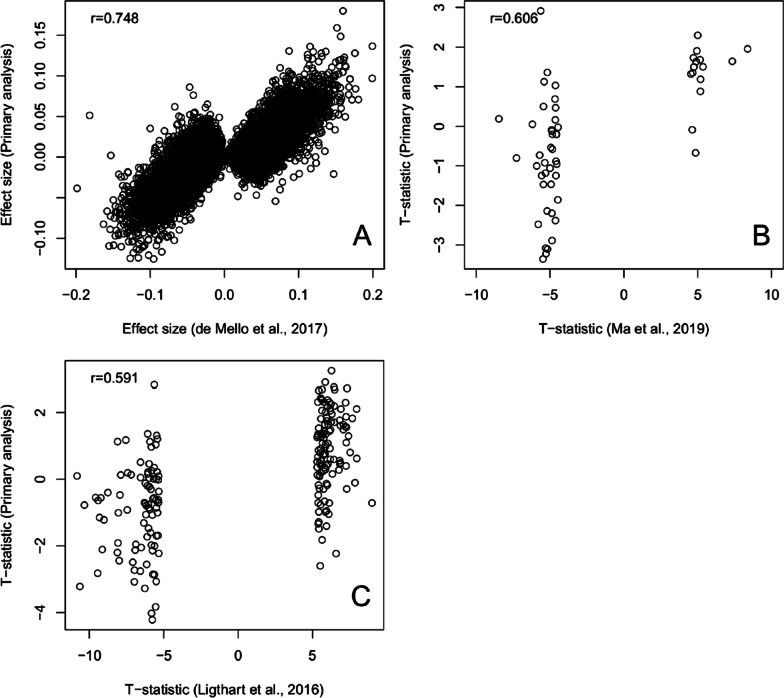
Comparisons with other analyses. **A** Effect sizes from our primary analysis compared to previous analyses. NASH-related EWAS in liver tissue. Test statistics from our primary analysis compared to **B** an EWAS investigating the association between hepatic fat accumulation. **C** An EWAS of inflammation (C-reactive protein) in blood

To further assess the robustness of the observed associations, we compared our results to a previous study that used the Illumina Infinium HumanMethylation450 BeadChip to analyze liver biopsies obtained from 95 obese individuals (35 with normal liver phenotype, 34 with simple steatosis, and 26 with NASH) [13]. A comparison of effect sizes between this study and the current work is shown in Fig. 5A and Additional file 1: Figure S7A. CpG-specific effect sizes from our study showed extremely high concordance with these previously published results (*r* = 0.75 and 0.94 when compared to our primary and secondary analysis), suggesting that a significant number of loci with changes in DNAm detected in NASH relative to normal histology may also be observed in fibrosis, which arises out of a background of NASH.

### DNA methylation profile of fibrosis correlates with test statistics from a blood-based study of hepatic fat

We next compared our EWAS results to those of a large (*N* = 1496) EWAS investigating the association between hepatic fat and DNAm in blood [14]. Their test statistics showed a correlation of *r* = 0.61 with the results of our primary analysis (Fig. 5B), and *r* = 0.28 with the results of our secondary analysis (Additional file 1: Fig S7B). These correlations support a partially shared DNAm signature of the two related NAFLD phenotypes, fibrosis and hepatic fat.

### Inflammation-related EWAS test statistics in blood are associated with liver fibrosis-related DNA methylation

Because NAFLD phenotypes including fibrosis severity [25] and hepatic fat [26] have previously shown mild association with levels of the inflammatory marker C-reactive protein (CRP), we compared our results to those of a large (*N* = 8863) EWAS investigating the association between CRP and DNAm in blood [27] to examine whether our results could reflect an inflammatory signature of DNAm. When comparing their test statistics to ours, we observed a correlation of *r* = 0.59 with our primary analysis (Fig. 5C) and *r* = 0.28 with our secondary analysis (Additional file 1: Fig S7C). The similarity of these correlations to the correlations observed with the hepatic fat DNAm signature suggests that inflammation may contribute to the DNAm signatures of NAFLD and related phenotypes.

### Associations between fibrosis-related DNA methylation and gene expression are consistent with trans-regulation

**Fig. 6:**
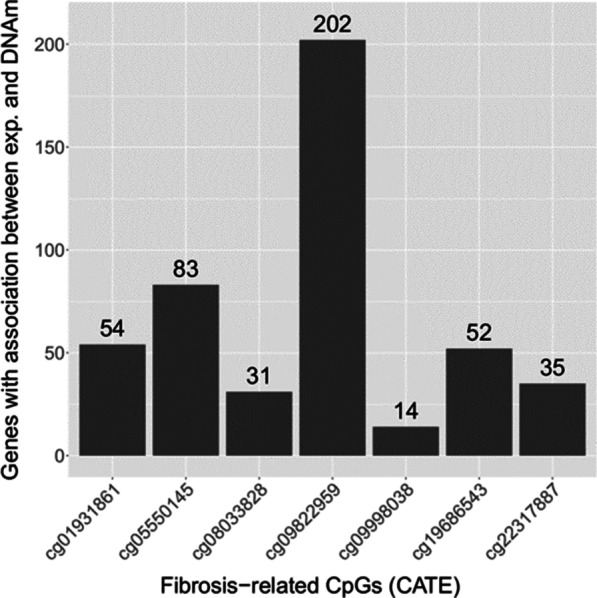
Number of genes with a significant association with DNAm across the 7 NAFLD-related CpGs

Association analysis of DNAm at the seven EWAS CpG sites compared to gene expression measured using RNA-seq was conducted using the R Bioconductor package DESeq2 [28]. Each of the fibrosis-related CpG sites was associated with differential gene expression of 14–202 genes (*p* < 3.2 × 10^–6^; Fig. 6). While most of the transcripts associated with DNAm at a single CpG site, some genes had significant associations with multiple CpG sites including nine genes with a significant association with DNAm at six of the CpG sites. Genes whose expression significantly associated with DNAm in at least four of the seven fibrosis-related CpG sites are listed in Additional file 1: Table S3. CpG sites and their associated genes were mostly located on different chromosomes, and all of them were separated by > 4 Mb, which may indicate a trans-acting effect (Additional file 1: Table S4).

We performed a separate GO analysis for each set of significant genes associated with CpG sites identified in Fig. 6. The gene sets corresponding to two of the CpG sites had significant GO terms (Additional file 1: Table S5). Twelve GO terms specifically mention morphogenesis. Curiously, six of the 12 terms were specific to heart morphogenesis. There were several other GO terms corresponding to processes involved in cardiovascular development.

We also performed a GO analysis to investigate whether the set of genes that associated with ≥ 3 NAFLD-related CpG sites were enriched for biological processes (Additional file 1: Table S6). Although no terms were significant, the term with the lowest *p* value was positive regulation of epithelial differentiation, which was one of the cell types we previously observed to change with disease progression based on EpiDISH estimates.

### DNA methylation is highly predictive of fibrosis

**Fig. 7:**
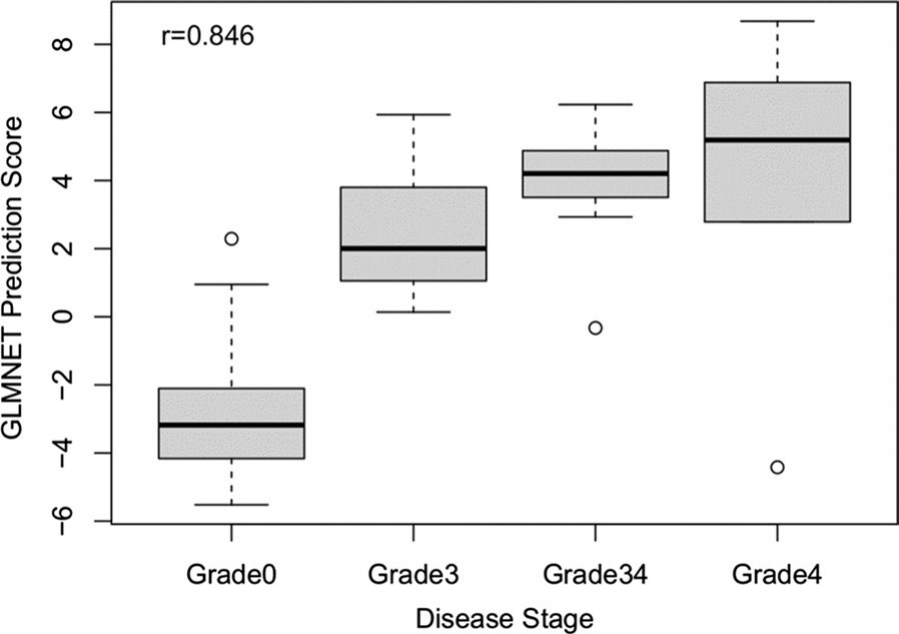
Prediction scores are highly correlated with NAFLD disease stage in independent test data

To assess whether DNAm can predict disease stage, we performed elastic net regression with tenfold cross-validation on an initial training set of DNAm data from 225 samples to tune hyperparameters *α* and *λ*, with the 100 remaining samples reserved for the independent test set. Elastic net regression adds the hyperparameters to minimize the size of all coefficients, including minimizing to zero and dropping predictor variables to avoid large estimated coefficients due to relatively fewer samples than input predictors or variables. As potential predictors in the model, we included the 15,000 most significant fibrosis-related CpG sites (*p* < 3.2 × 10^–40^) from the covariate-adjusted linear regression model. Our resulting model included 28 CpGs (Additional file 1: Table S7) and had a test accuracy of 94% with a sensitivity of 93.1% (27 of 29 true positives) and a specificity of 94.4% (67 of 71 true negatives) (Table 4). The prediction score provided by our model was highly correlated with fibrosis in our test set (*r* = 0.846, Fig. 7).

**Table 4:** Prediction of elastic net model across the four observed grades of NAFLD

	Predicted healthy	Predicted fibrosis
Grade 0	67	4
Grade 3	0	12
Grade 3/4	1	10
Grade 4	1	5

Hyperparameters: *α* = 1, *λ* = 0.025

Additional file 1: Figure S8 shows that the prediction score was associated with estimated proportions of every cell type, except for monocytes. To assess whether our predictive power mainly reflected differences in cell-type proportions, we also performed an elastic net regression with tenfold cross-validation using EpiDISH-estimated cell-type proportions and observed an accuracy of 79%. This indicates that a model tuned using raw DNAm has greater potential as a biomarker of NAFLD fibrosis than a model based simply on the estimated cell-type proportions.

## Discussion

Most studies of DNAm in human NAFLD have been focused on candidate CpG sites and have analyzed pre-fibrotic stages of NAFLD using a restricted number of samples. We performed an EWAS of severe fibrosis in NAFLD with a larger sample size (*n* = 325) than has yet been reported. In comparison with our EWAS results to those from EWAS of NAFLD and hepatic fat, we observed high concordance between results. Since hepatic fat accumulation is a phenotype related to NAFLD, this comparison may indicate that DNAm regulates the same genes in both blood and liver throughout NAFLD pathogenesis. The observed high concordance between both our subgroup analyses and our results and previously published findings further suggests that the observed DNAm signature of fibrosis is robust.

Interestingly, our EWAS results in liver tissue were positively correlated with results from an inflammation-related EWAS in blood. Association between DNAm signatures of NAFLD fibrosis and CRP is consistent with previous work that reported increased CRP levels in NAFLD patients [25, 26]. These results may suggest that the DNAm signature associated with fibrosis partially reflects increased inflammation or that the same inflammation-related genes regulated by blood DNAm are also regulated by liver DNAm in NAFLD.

A well-known issue in blood-based EWAS is that different components of whole blood often have distinct DNAm profiles [29, 30]. Because cell-type composition often differs between disease states, it is not always apparent whether an association detected in an EWAS reflects a true difference in DNAm or merely a difference in cell-type composition. There is no one-size-fits-all method for correcting for cell-type composition in EWAS analyses, and as Teschendorff and Zheng [29] point out, the best method to address this issue may depend on the tissue type and phenotype being investigated. When investigating the relationship between DNAm in liver and NAFLD fibrosis, cell composition is of particular importance considering its critical role in NAFLD pathogenesis, including the differentiation of hepatic stellate cells to myofibroblasts and infiltration of immune cells [31]. To account for cell composition in our primary analysis, we employed a reference-free method [32] that estimates and adjusts for latent factors in high-dimensional data. Using this approach, we observed seven hypomethylated CpG sites. For comparison, we observed tens of thousands of associated CpG sites in unadjusted linear models (257,825 sites) or in those adjusted for the cell-type proportions estimated via EpiDISH (58,686 sites). The reduced number of significant results in the primary analysis compared to the analysis with EpiDISH-estimated cell types may indicate that the cellular heterogeneity was not adequately captured by the reference used. Reference-free methods are capable of fully adjusting for genomic inflation. In contrast, a reference-based method can adequately adjust for genomic inflation only if the reference sets fully capture the distinct DNAm signatures for each cell type. This task can be challenging if a tissue has many cell subsets each with distinct DNAm signatures. For example, Diedrich et al. [33] observed decreased frequencies of CD8^+^ T cells, Vd2^+^γδ T cells, and CD56_bright_ NK cells, but increased frequencies of Vδ2^−^γδ T cells and CD56_dim_ NK cells in liver samples in NAFLD patients compared to controls. Moreover, complex patterns underlie the NAFLD-related cell differentiation and migration, which could affect the accuracy of a method attempting to deconvolute cell types. Krenkel et al. [22] found that myofibroblasts split into functionally distinct heterogeneous populations of cells in fibrosis-induced mice, both in vivo and in vitro. Furthermore, a study investigating transcriptomes in 100,000 human cells found that cirrhotic human liver tissue is characterized by a pro-fibrogenic subpopulation of macrophages, as well as endothelial cell subsets that enhance the transmigration of leukocytes [21]. Thus, one of the limitations of this study is that cell composition is a potential confounder in epigenome-wide association studies, particularly when the DNAm is extracted from composite tissues. Because there is not yet a widely agreed upon method to adjust for cell composition, we performed our primary analysis adjusted for latent confounders, followed by a secondary analysis adjusting for EpiDISH-estimated cell types.

Nevertheless, our comparison with the results from a recent EWAS of hepatic fat and blood DNAm [14] suggests that our inclusion of latent confounders was correcting for cell composition. We observed a significant correlation between the two studies; notably, the results from the hepatic fat EWAS showed a much higher correlation (*r* = 0.61 compared to *r* = 0.28) with our primary analysis adjusted for latent confounders than with our secondary analysis (Fig. 5B). Considering that we are investigating related phenotypes (hepatic fat accumulation vs NASH fibrosis) in distinct tissues (blood vs liver), this increase in correlation may indicate that the reference-free primary analysis is successfully adjusting for cell composition.

As a complement to DNAm, single-cell RNA sequencing (scRNA-seq) has the potential to measure cell composition at a higher resolution and could facilitate greater elucidation of the relationship between NAFLD fibrosis, cellular composition, and DNAm. Many methods have been developed to utilize scRNA-seq data to cluster cells into their respective cell types [34, 35]. Such methods could potentially be combined with DNAm studies considering that a previous study has been able to capture RNA-sequencing data and bisulfite-sequencing data within the same cells [36]. However, there are currently many more methods that utilize scRNA-seq data to cluster cells into their respective cell types than there are ways to perform similar clustering using single-cell DNAm data. It may be possible to infer cell type to a high accuracy from single-cell DNAm data alone considering that DNAm profiles can be specific to cell lineage [37]. A method called RETrace estimates cell-type proportions from DNAm measured using single cell reduced representation bisulfite sequencing [38].

We performed gene ontology analysis on both the EWAS results and downstream RNA-seq results, and while there were no significant GO terms in the primary analysis, GO terms with the lowest *p* values were related to apoptosis and morphogenesis, both of which occur in NAFLD [39, 40]. Based on the GO analysis of genes associated with ≥ 4 CpG sites (Additional file 1: Table S5), some of the top 35 GO terms (0.05 < FDR < 0.1) corresponded to processes involved in NAFLD fibrosis, including three terms corresponding to apoptosis and two terms corresponding to epithelial cell differentiation. The most significant GO term corresponded to B cell receptor transport. Although the role of B cells in NAFLD is not as well-characterized as other immune cell types, recent work suggests this cell type is involved in hepatic fat accumulation [41]. Considering this GO analysis was performed on genes associated with DNAm at multiple CpG sites, it is possible that these biological processes involved in NAFLD are orchestrated via the coordinated regulation of gene expression across multiple CpG sites.

Our elastic net regression model demonstrates that DNAm may have potential as a biomarker to diagnose NAFLD. Our model was able to predict NAFLD fibrosis with a 94% accuracy in our test set with a sensitivity of 93.1% and a specificity of 94.4%. A potential drawback of our model is that it was performed in liver tissue, which is relatively invasive to obtain, especially compared to blood. Thus, a limitation of this analysis is that we were unable to perform it on blood-based DNAm, which would be a less-invasive candidate tissue for a noninvasive diagnostic tool. However, results from our primary and secondary analyses were strongly associated with results from previous work investigating blood DNAm related to hepatic fat accumulation [14]. These results suggest that our model could potentially predict NAFLD fibrosis in blood DNAm. Circulating DNAm has been suggested as a potential noninvasive biomarker of disease severity for NAFLD [42, 43]. Hardy et al. [10] observed an association between plasma DNAm in the PPARγ gene promoter and severity of fibrosis in 26 NAFLD patients, ranging from mild (F0–F2) to severe fibrosis (F3–F4), and reported that an optimal cutoff of 81% DNAm was able to separate NAFLD patients with mild versus severe fibrosis with a sensitivity of 83% and a specificity of 93%, though prediction accuracy was not assessed in an independent dataset. The same research group also observed an association between DNAm and fibrosis in the PPARγ promoter in a Turkish cohort [44]. Using DNAm from blood leukocyte samples, Wu et al. [15] also searched for optimal cutoffs in several genes to separate NASH patients from NAFLD patients with simple hepatic steatosis. However, to date, the accuracy of DNAm to predict NAFLD disease state has not been assessed in an independent dataset.

Here, we investigated the DNAm profile of advanced fibrosis with approximately twice as many CpG sites and more than three times the number of samples compared to previous work. Using the DNAm data, we showed that estimated levels of epithelial cells decrease, while levels of immune cells increase, with fibrosis severity, suggesting that shifts in cell composition may partially explain changes in DNAm observed across fibrotic stage. In addition to contributing to a better understanding of the underlying biology of NAFLD fibrosis, we created a DNAm-based model capable of predicting fibrotic stage with very high accuracy. Overall, our investigation shows that DNAm provides information that is not only useful for understanding the underlying biology of NAFLD, but may also serve as a clinical tool capable of independently diagnosing fibrosis.

## Methods

### Study participants

Liver wedge biopsies were intraoperatively obtained from individuals enrolled in the Bariatric Surgery Program at the Geisinger Clinic Center for Nutrition and Weight Management and histologically evaluated using NASH CRN criteria as described [45]. Patients with histologic or serologic evidence for other chronic liver diseases were excluded from this study. Both medical history and histological assessment excluded individuals with clinically significant alcohol intake and drug use from participation in the bariatric surgery program. Clinical data including demographics, clinical measures, ICD-9 codes, medical history, medication use, and common laboratory results were available for all study participants as described previously [46].

### DNA methylation analysis

Frozen liver biopsy specimens were minced and lysed using the Bullet Blender Bead Lysis kit (Next Advance; Troy, NY). Liver genomic DNA was extracted with the ALLprep DNA/RNA Mini Kit (Qiagen; Valencia, CA) and quantified using the Qubit system (ThermoFisher Scientific; Waltham, MA). Whole genome DNAm profiling was performed using the Infinium MethylationEPIC BeadChip kit (Illumina; San Diego, CA). We used the EZ DNA Methylation Kit (Zymo Research; Irvine, CA) for sodium bisulfite conversion of DNA, and bisulfite-treated DNA was fragmented and hybridized to the bead chips. Bead chips were scanned and imaged with the iScan system (Illumina). DNAm levels for each CpG residue were estimated as the ratio of the methylated signal intensity over the sum of the methylated and unmethylated intensities at each locus.

### DNA methylation data processing

We performed background correction using the preprocessNoob function with default settings in the R package minfi [47, 48]. We then removed samples with > 5% missingness and CpGs with > 5% missingness, as well as CpGs with a detection *p* value > 0.001 using the cpg.qc() function in the R package CpGassoc [49].

We performed several diagnostic tests to check for outliers or potential sample swaps. To check for sample swaps, we computed DNAm-inferred age [50] and DNAm-inferred sex on the samples. We then regressed chronological age on the DNAm-inferred age and removed any samples whose residuals exceeded 15 (indicating a roughly 15-year difference in DNAm-predicted age vs. recorded age, and thus a possible sample swap). We also excluded any samples whose observed sex differed from DNAm-inferred sex.

We used principal components analysis (PCA) on the matrix of DNAm β-values to check for outliers and assess concordance between technical replicates. Intentional duplicates were included for multiple samples including one technical replicate derived from a single sample with 8 runs, and 11 samples each with 2 duplicate runs. After removing all potential outliers/sample swaps, we averaged the *β* values for each CpG site for the replicates and duplicates within their respective samples.

### Estimation of cell-type proportions

We used EpiDISH, a reference-based method of estimating cell-type proportions based on genome-wide DNAm data [24] to estimate proportions of each cell type. EpiDISH uses two non-overlapping references to estimate cell-type proportions. The first reference enables estimation of proportions of fibroblast cells, epithelial cells, and immune cells, while the second reference allows further deconvolution of the immune cells into NK cells, B cells, and monocytes. We performed analyses using both references.

### Epigenome-wide association analyses

For our primary analysis, we performed an epigenome-wide association study (EWAS) using the R package CATE [32] using the robust regression adjustment method with 2 latent confounders to allow for adjustment of unobserved confounders including technical factors and variation in cell-type proportions. DNAm *β* values were modeled as the dependent variable, while fibrosis status (yes/no) was the independent variable of interest with age, sex, and BMI included as covariates. To further interpret and assess robustness of our results, we also performed several secondary sensitivity analyses, including a similar EWAS where NAFLD patients with no fibrosis (F0) were compared to those with cirrhosis (F4) only.

For comparison to our primary analysis that adjusted for latent confounders, we performed a secondary analysis considering an alternative model that directly adjusted for potential confounders as covariates. For this analysis, we used the R package CpGassoc to fit mixed effects linear models with DNAm *β* values as the dependent variable, fibrosis status as the independent variable, and age, sex, BMI, and cell-type proportions as covariates, and chip ID as a random effect. For all analyses, we used a Bonferroni correction ($p < \frac{0.05}{{839,596}}$) to correct for the number of CpG sites tested.

### Overlap of NAFLD-related DNAm with enhancers

We investigated the overlap of CpGs with the NAFLD-related DNAm in the primary analysis to an annotation of enhancers [51]. In addition to reporting on the overlaps with enhancers, we also reported on any genes linked to those enhancers.

### Enrichment analyses

We used the R/Bioconductor package missMethyl to test whether fibrosis-related CpG sites were enriched for biological pathways [52]. We performed a separate enrichment analysis for each EWAS performed above. To test for enrichment of genes whose expression associated with DNAm at NAFLD-related CpG sites, we used the GOStats R/Bioconductor package [53].

### Comparisons with previous analyses

To investigate the concordance of fibrosis-related DNAm with previous work, we compared the effect sizes of our EWAS analyses (both the primary and secondary analyses) to those reported in a previous EWAS of NASH in liver samples [13]. We also compared our results to a large EWAS of hepatic fat based on 1496 blood samples [14]. Finally, we compared our findings to a previous study investigating genome-wide inflammation-related DNAm based on a discovery cohort of *N* = 8863 [27]. For all of these comparisons, we compared test statistics from our primary and secondary analyses to previously published test statistics by calculating Pearson’s correlation coefficient to assess similarity of direction and magnitude of effects.

### Comparison of gene expression and DNA methylation

RNA-seq was performed to measure genome-wide gene expression in 56 of the 325 individuals in the EWAS as described [54]. We first aligned paired-end RNA-seq fastq files to the hg38 build of the human genome. Genes with fewer than 10 reads in ≥ 14 samples were excluded from the analysis. We then used the R Bioconductor packages Rsamtools [55] and Genomic Ranges [56] in the hg38 build of the human genome. We used the same hg38 gene annotation file (Homo_sapiens.GRCh38.87.gtf) to build the STAR genome index and the count matrix. Using the DESeq2 package [28], we tested for an association between gene expression and DNAm, where read counts for each transcript were modeled as a function of CpG-specific DNAm, controlling for sex, age, BMI, and estimated cell-type proportions using both EpiDISH references. We used a Bonferroni cutoff ($p < \frac{0.05}{{15,414}}$) to correct for multiple testing.

### Elastic net regression model to predict disease status in NAFLD

To predict fibrosis status, we performed an elastic net regression with tenfold cross-validation using the glmnet and caret packages in R [57]. For potential predictors, we used DNAm from the top 15,000 NAFLD-related CpGs from the linear model unadjusted for cell types. The outcome variable was fibrosis disease status (yes/no).

## Supplementary Information

**Additional file 1**. Supplementary materials.

## Data Availability

The matrix of DNA methylation beta values and phenotypic and demographic information is available on the Gene Expression Omnibus (https://www.ncbi.nlm.nih.gov/geo; accession number GSE180474). Raw RNA-seq reads are available at the Sequence Read archive (https://www.ncbi.nlm.nih.gov/sra; accession number PRJNA512027).

## References

[1] Li B, Zhang C, Zhan YT (2018). Nonalcoholic fatty liver disease cirrhosis: a review of its epidemiology, risk factors, clinical presentation, diagnosis, management, and prognosis. Can J Gastroenterol Hepatol.

[2] Dowson C, O’Reilly S (2016). DNA methylation in fibrosis. Eur J Cell Biol.

[3] Benedict M, Zhang X (2017). Non-alcoholic fatty liver disease: an expanded review. World J Hepatol.

[4] McCullough AJ (2004). The clinical features, diagnosis and natural history of nonalcoholic fatty liver disease. Clin Liver Dis.

[5] Pennisi G, Celsa C, Giammanco A, Spatola F, Petta S (2019). The burden of hepatocellular carcinoma in non-alcoholic fatty liver disease: screening issue and future perspectives. Int J Mol Sci.

[6] Hyun J, Jung Y (2020). DNA methylation in nonalcoholic fatty liver disease. Int J Mol Sci.

[7] Papait R, Serio S, Condorelli G (2020). Role of the epigenome in heart failure. Physiol Rev.

[8] Chen L, Huang W, Wang L, Zhang Z, Zhang F, Zheng S (2020). The effects of epigenetic modification on the occurrence and progression of liver diseases and the involved mechanism. Expert Rev Gastroenterol Hepatol.

[9] Götze S, Schumacher EC, Kordes C, Häussinger D (2015). Epigenetic changes during hepatic stellate cell activation. PLoS ONE.

[10] Hardy T, Zeybel M, Day CP, Dipper C, Masson S, McPherson S (2017). Plasma DNA methylation: a potential biomarker for stratification of liver fibrosis in non-alcoholic fatty liver disease. Gut.

[11] Zeybel M, Hardy T, Robinson SM, Fox C, Anstee QM, Ness T (2015). Differential DNA methylation of genes involved in fibrosis progression in non-alcoholic fatty liver disease and alcoholic liver disease. Clin Epigenetics.

[12] Gerhard GS, Malenica I, Llaci L, Chu X, Petrick AT, Still CD (2018). Differentially methylated loci in NAFLD cirrhosis are associated with key signaling pathways. Clin Epigenetics.

[13] de Mello VD, Matte A, Perfilyev A, Mannisto V, Ronn T, Nilsson E (2017). Human liver epigenetic alterations in non-alcoholic steatohepatitis are related to insulin action. Epigenetics.

[14] Ma J, Nano J, Ding J, Zheng Y, Hennein R, Liu C (2019). A peripheral blood DNA methylation signature of hepatic fat reveals a potential causal pathway for nonalcoholic fatty liver disease. Diabetes.

[15] Wu J, Zhang R, Shen F, Yang R, Zhou D, Cao H (2018). Altered DNA methylation sites in peripheral blood leukocytes from patients with simple steatosis and nonalcoholic steatohepatitis (NASH). Med Sci Monit.

[16] Ahrens M, Ammerpohl O, von Schönfels W, Kolarova J, Bens S, Itzel T (2013). DNA methylation analysis in nonalcoholic fatty liver disease suggests distinct disease-specific and remodeling signatures after bariatric surgery. Cell Metab.

[17] Hotta K, Kitamoto A, Kitamoto T, Ogawa Y, Honda Y, Kessoku T (2018). Identification of differentially methylated region (DMR) networks associated with progression of nonalcoholic fatty liver disease. Sci Rep.

[18] Murphy SK, Yang H, Moylan CA, Pang H, Dellinger A, Abdelmalek MF (2013). Relationship between methylome and transcriptome in patients with nonalcoholic fatty liver disease. Gastroenterology.

[19] Decamps C, Privé F, Bacher R, Jost D, Waguet A, Houseman EA (2020). Guidelines for cell-type heterogeneity quantification based on a comparative analysis of reference-free DNA methylation deconvolution software. BMC Bioinform.

[20] Dobie R, Wilson-Kanamori JR, Henderson BEP, Smith JR, Matchett KP, Portman JR (2019). Single-cell transcriptomics uncovers zonation of function in the mesenchyme during liver fibrosis. Cell Rep.

[21] Ramachandran P, Dobie R, Wilson-Kanamori JR, Dora EF, Henderson BEP, Luu NT (2019). Resolving the fibrotic niche of human liver cirrhosis at single-cell level. Nature.

[22] Krenkel O, Hundertmark J, Ritz TP, Weiskirchen R, Tacke F (2019). Single cell RNA sequencing identifies subsets of hepatic stellate cells and myofibroblasts in liver fibrosis. Cells.

[23] Silva JP, van Booven D (2018). Analysis of diet-induced differential methylation, expression, and interactions of lncRNA and protein-coding genes in mouse liver. Sci Rep.

[24] Teschendorff AE, Breeze CE, Zheng SC, Beck S (2017). A comparison of reference-based algorithms for correcting cell-type heterogeneity in Epigenome-Wide Association Studies. BMC Bioinform.

[25] Yoneda M, Mawatari H, Fujita K, Iida H, Yonemitsu K, Kato S (2007). High-sensitivity C-reactive protein is an independent clinical feature of nonalcoholic steatohepatitis (NASH) and also of the severity of fibrosis in NASH. J Gastroenterol.

[26] Fricker ZP, Pedley A, Massaro JM, Vasan RS, Hoffmann U, Benjamin EJ (2019). Liver fat is associated with markers of inflammation and oxidative stress in analysis of data from the Framingham heart study. Clin Gastroenterol Hepatol.

[27] Ligthart S, Marzi C, Aslibekyan S, Mendelson MM, Conneely KN, Tanaka T (2016). DNA methylation signatures of chronic low-grade inflammation are associated with complex diseases. Genome Biol.

[28] Love MI, Huber W, Anders S (2014). Moderated estimation of fold change and dispersion for RNA-seq data with DESeq2. Genome Biol.

[29] Teschendorff AE, Zheng SC (2017). Cell-type deconvolution in epigenome-wide association studies: a review and recommendations. Epigenomics.

[30] Koestler DC, Christensen B, Karagas MR, Marsit CJ, Langevin SM, Kelsey KT (2013). Blood-based profiles of DNA methylation predict the underlying distribution of cell types: a validation analysis. Epigenetics.

[31] Jou J, Choi SS, Diehl AM (2008). Mechanisms of disease progression in nonalcoholic fatty liver disease. Semin Liver Dis.

[32] Wang J, Zhao Q, Hastie T, Owen AB (2017). Confounder adjustment in multiple hypothesis testing. Ann Stat.

[33] Diedrich T, Kummer S, Galante A, Drolz A, Schlicker V, Lohse AW (2020). Characterization of the immune cell landscape of patients with NAFLD. PLoS ONE.

[34] Stuart T, Satija R (2019). Integrative single-cell analysis. Nat Rev Genet.

[35] Abdelaal T, Michielsen L, Cats D, Hoogduin D, Mei H, Reinders MJT (2019). A comparison of automatic cell identification methods for single-cell RNA sequencing data. Genome Biol.

[36] Gaiti F, Chaligne R, Gu H, Brand RM, Kothen-Hill S, Schulman RC (2019). Epigenetic evolution and lineage histories of chronic lymphocytic leukaemia. Nature.

[37] Salas LA, Wiencke JK, Koestler DC, Zhang Z, Christensen BC, Kelsey KT (2018). Tracing human stem cell lineage during development using DNA methylation. Genome Res.

[38] Wei CJ, Zhang K (2020). RETrace: simultaneous retrospective lineage tracing and methylation profiling of single cells. Genome Res.

[39] Alkhouri N, Carter-Kent C, Feldstein AE (2011). Apoptosis in nonalcoholic fatty liver disease: diagnostic and therapeutic implications. Expert Rev Gastroenterol Hepatol.

[40] Lotowska JM, Sobaniec-Lotowska ME, Lebensztejn DM (2013). The role of Kupffer cells in the morphogenesis of nonalcoholic steatohepatitis—ultrastructural findings. The first report in pediatric patients. Scand J Gastroenterol.

[41] Koo SY, Park EJ, Lee CW (2020). Immunological distinctions between nonalcoholic steatohepatitis and hepatocellular carcinoma. Exp Mol Med.

[42] Moran-Salvador E, Mann J (2017). Epigenetics and liver fibrosis. Cell Mol Gastroenterol Hepatol.

[43] Perakakis N, Stefanakis K, Mantzoros CS (2020). The role of omics in the pathophysiology, diagnosis and treatment of non-alcoholic fatty liver disease. Metabolism.

[44] Yiğit B, Boyle M, Özler O, Erden N, Tutucu F, Hardy T (2018). Plasma cell-free DNA methylation: a liquid biomarker of hepatic fibrosis. Gut.

[45] DiStefano JK, Kingsley C, Craig Wood G, Chu X, Argyropoulos G, Still CD (2015). Genome-wide analysis of hepatic lipid content in extreme obesity. Acta Diabetol.

[46] Wood GC, Chu X, Manney C, Strodel W, Petrick A, Gabrielsen J (2012). An electronic health record-enabled obesity database. BMC Med Inform Decis Mak.

[47] Aryee MJ, Jaffe AE, Corrada-Bravo H, Ladd-Acosta C, Feinberg AP, Hansen KD (2014). Minfi: a flexible and comprehensive Bioconductor package for the analysis of Infinium DNA methylation microarrays. Bioinformatics.

[48] Triche TJ, Weisenberger DJ, Van Den Berg D, Laird PW, Siegmund KD (2013). Low-level processing of illumina infinium DNA methylation beadarrays. Nucleic Acids Res.

[49] Barfield RT, Kilaru V, Smith AK, Conneely KN (2012). CpGassoc: an R function for analysis of DNA methylation microarray data. Bioinformatics.

[50] Horvath S (2013). DNA methylation age of human tissues and cell types. Genome Biol.

[51] Fishilevich S, Nudel R, Rappaport N, Hadar R, Plaschkes I, Iny Stein T (2017). GeneHancer: genome-wide integration of enhancers and target genes in GeneCards. Database (Oxford).

[52] Phipson B, Maksimovic J, Oshlack A (2016). missMethyl: an R package for analyzing data from Illumina’s HumanMethylation450 platform. Bioinformatics.

[53] Falcon S, Gentleman R (2007). Using GOstats to test gene lists for GO term association. Bioinformatics.

[54] Gerhard GS, Legendre C, Still CD, Chu X, Petrick A, DiStefano JK (2018). Transcriptomic profiling of obesity-related nonalcoholic steatohepatitis reveals a core set of fibrosis-specific genes. J Endocr Soc.

[55] Morgan M, Pagès H, Obenchain V, Hayden N. Rsamtools: binary alignment (BAM), FASTA, Variant Call (BCF), and Tabix File Import. 2018.

[56] Lawrence M, Huber W, Pagès H, Aboyoun P, Carlson M, Gentleman R (2013). Software for computing and annotating genomic ranges. PLoS Comput Biol.

[57] Kuhn M (2008). Building predictive models in R using the caret package. J Stat Softw.

