# Supplementary material for: Differential DNA methylation and changing cell-type proportions as fibrotic stage progresses in NAFLD
Source: Clin Epigenetics. 2021 Aug 5;13:152. doi: 10.1186/s13148-021-01129-y (PMC8340447; doi:10.1186/s13148-021-01129-y)
Supplement: Supplementary file 1 — Additional file 1. Supplementary materials. [file 13148_2021_1129_MOESM1_ESM.docx]

**Supplementary material**

**
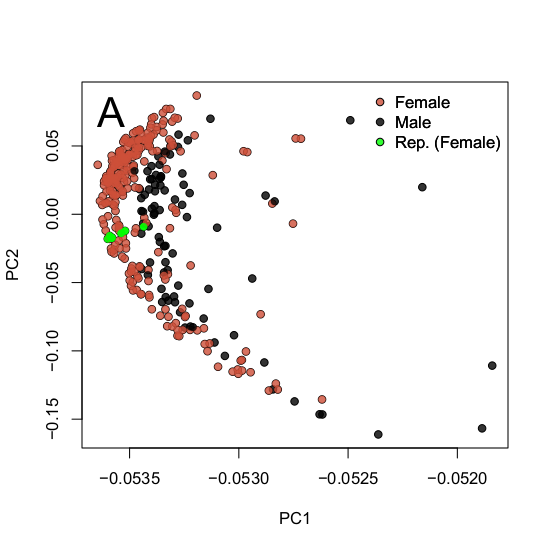

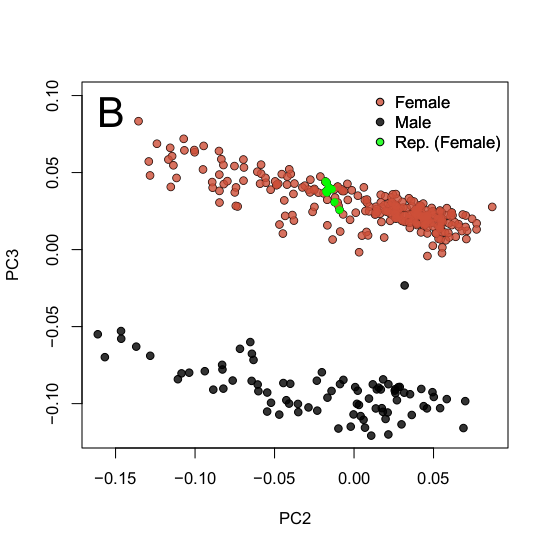

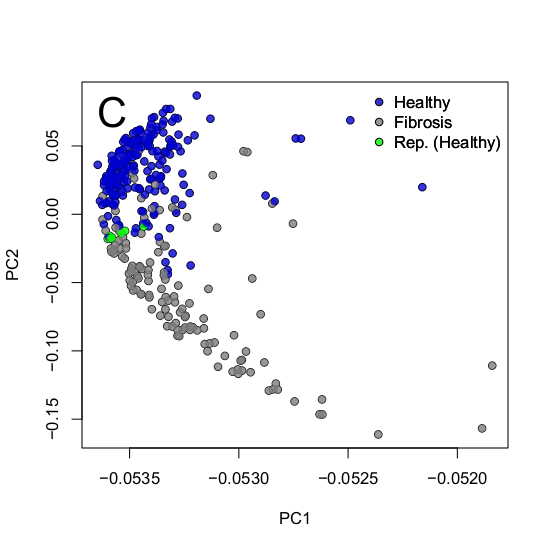

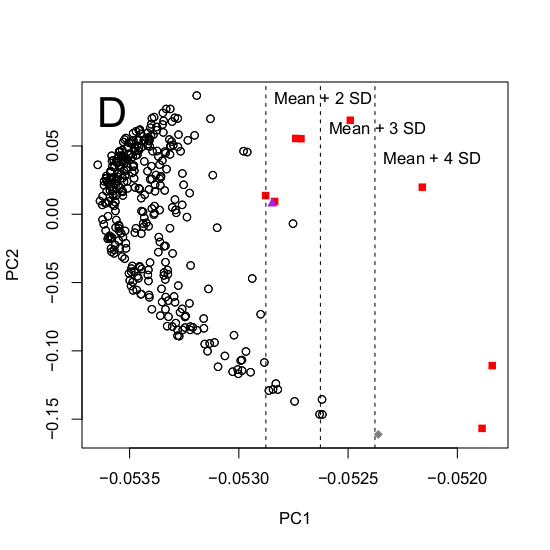
**

**Figure S1.** PCA plots color-coded by phenotype. Panel A is PC 2 vs. PC 1 color-coded by sex whereas Panel B is similarly color-coded by sex depicting PC 3 vs. PC 2. In Panel C, PC 2 is plotted against PC 1 color-coded by disease state. Panel D depicts outliers removed from the analysis. One of the chips comprised the majority of outliers for principal component (PC) 1, with PC 1 more than 2 standard deviations (SD) away from the mean for all 8 samples (in red); samples on this chip were excluded from subsequent analyses. Further, we excluded a duplicate where PC 1 was >2 SD from the mean (depicted as a purple diamond) as well as a non-duplicated sample where PC 1 was >4 SD from the mean (depicted as a grey triangle) (Figure PCA).


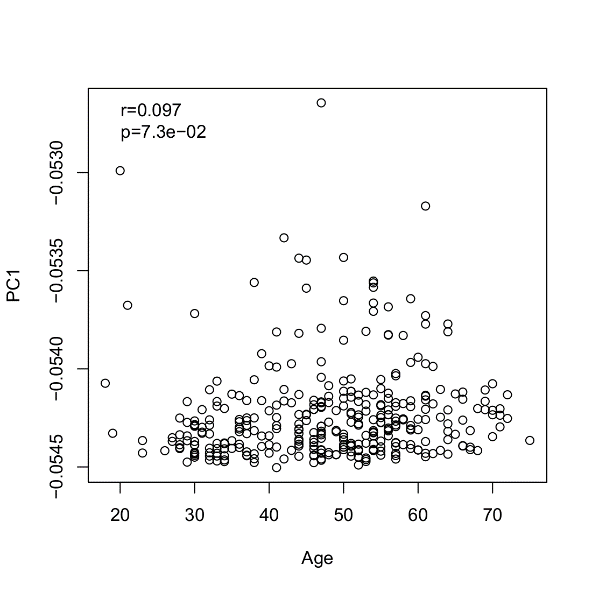

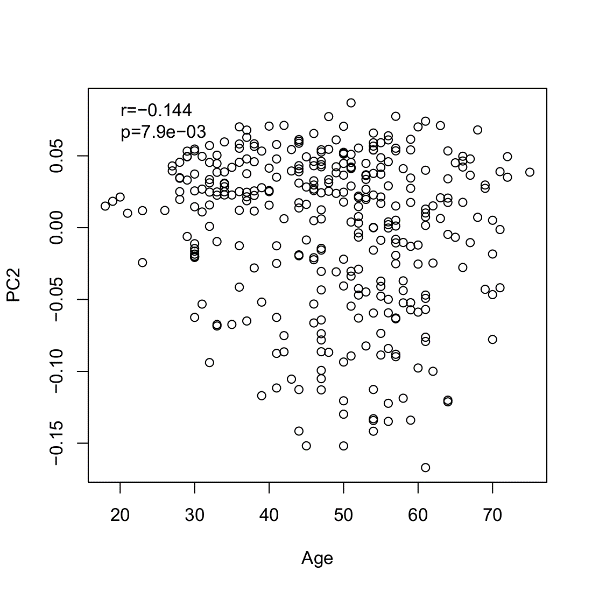

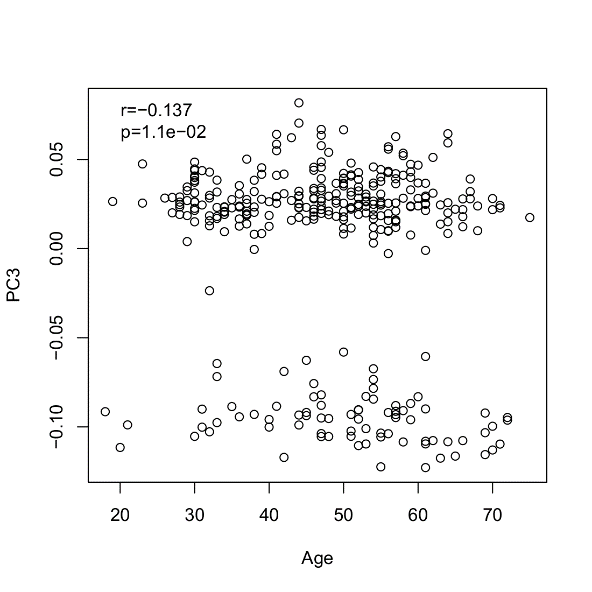

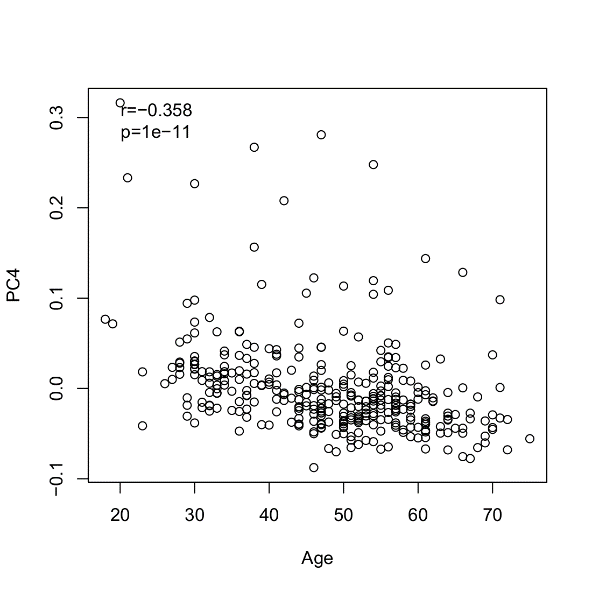

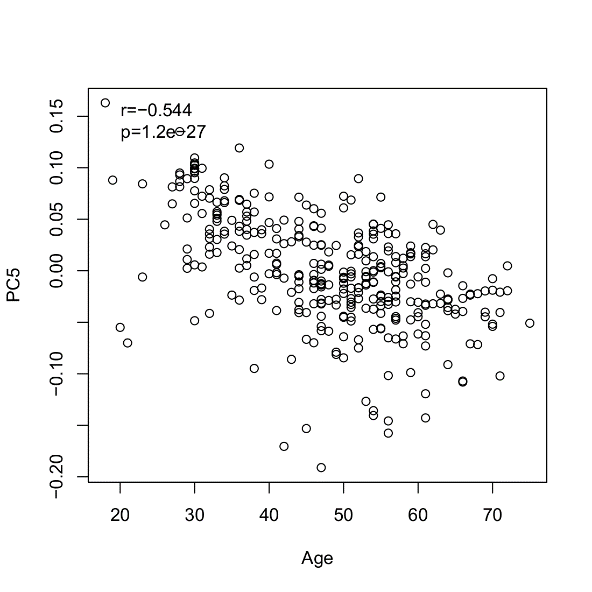

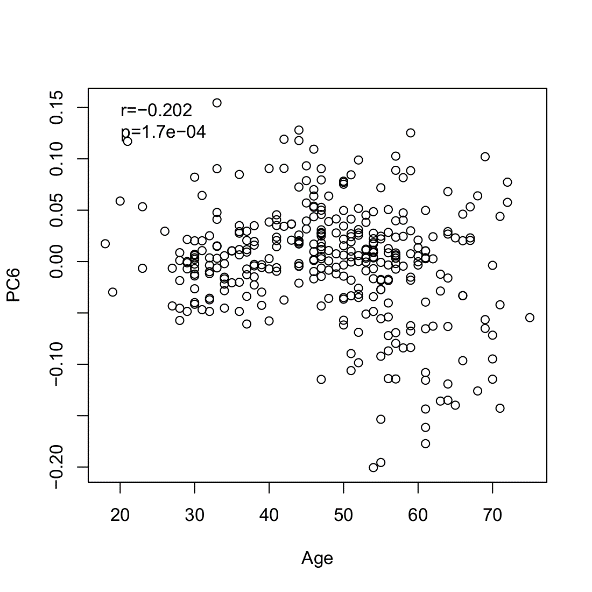

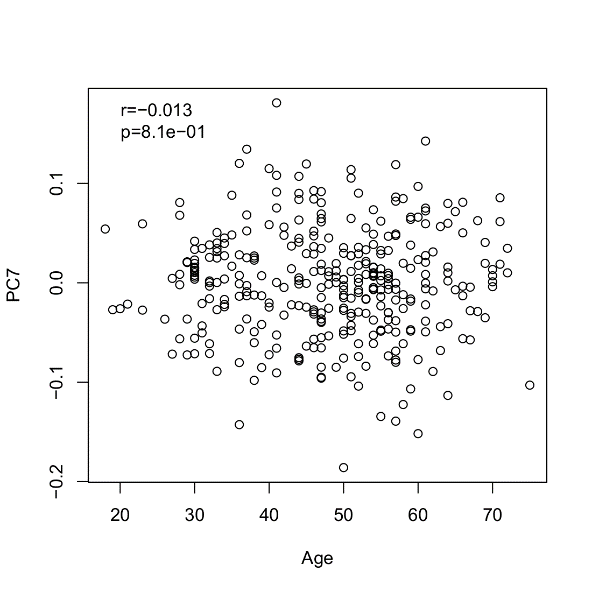

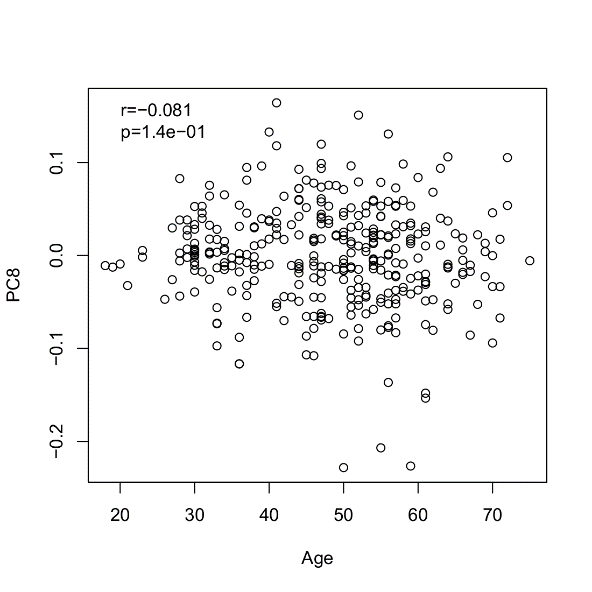

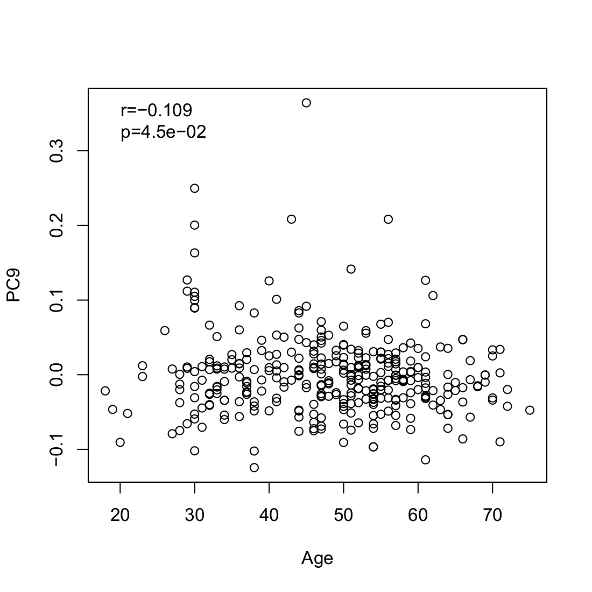

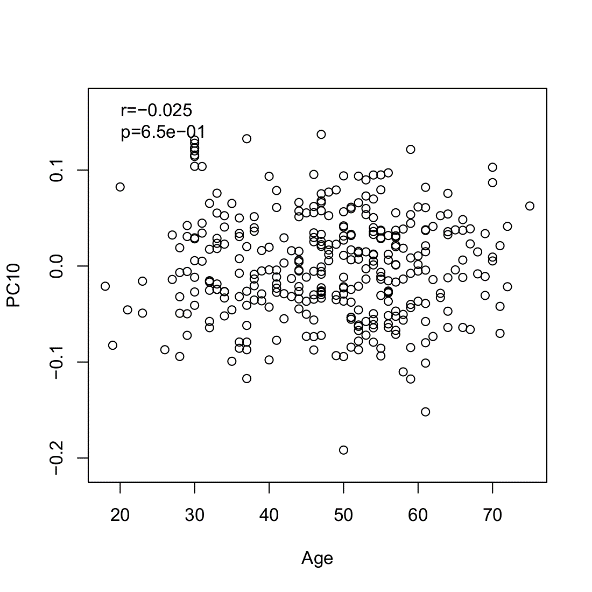


**Figure S2.** Principal components (PC1-PC10) plotted against chronological age. PC2-PC6 and PC9 were all significantly associated with age.

**
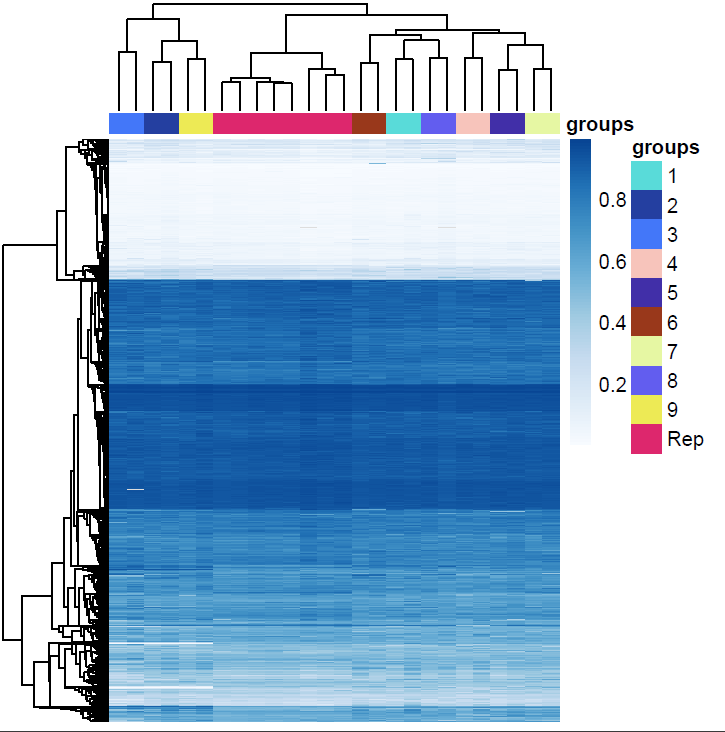
**

**Figure S3.** 9 samples with 2 duplicates each indicated by color as well as a sample with 8 replicates. Duplicates and replicates cluster by sample.


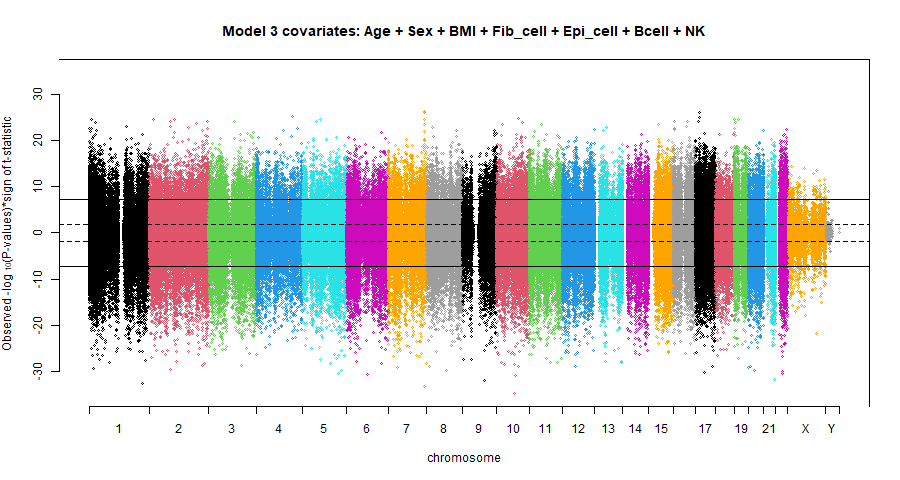


**Figure S4.** In the linear model adjusting for cell composition using two EpiDISH references, 25,667 CpG sites were hypermethylated and 33,411 CpG sites were hypomethylated.

**
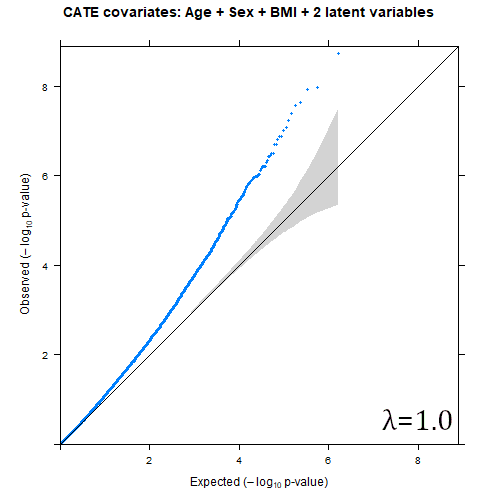

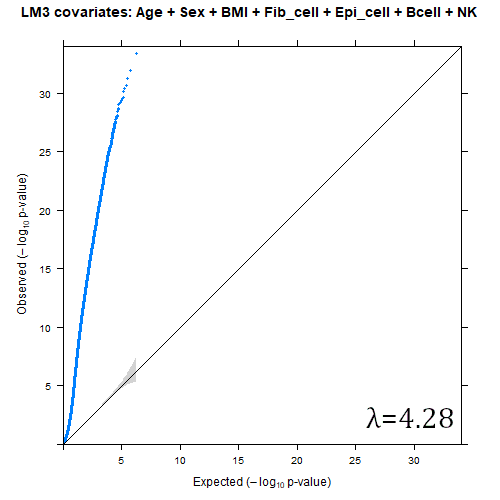
**

**Figure S5.** QQ plots of the primary analysis (left) and the analysis adjusting for EpiDISH-inferred cell composition (right). The genomic inflation factor is λ=1.0 for the primary analysis and λ=4.28 for the analysis adjusting for cell composition.


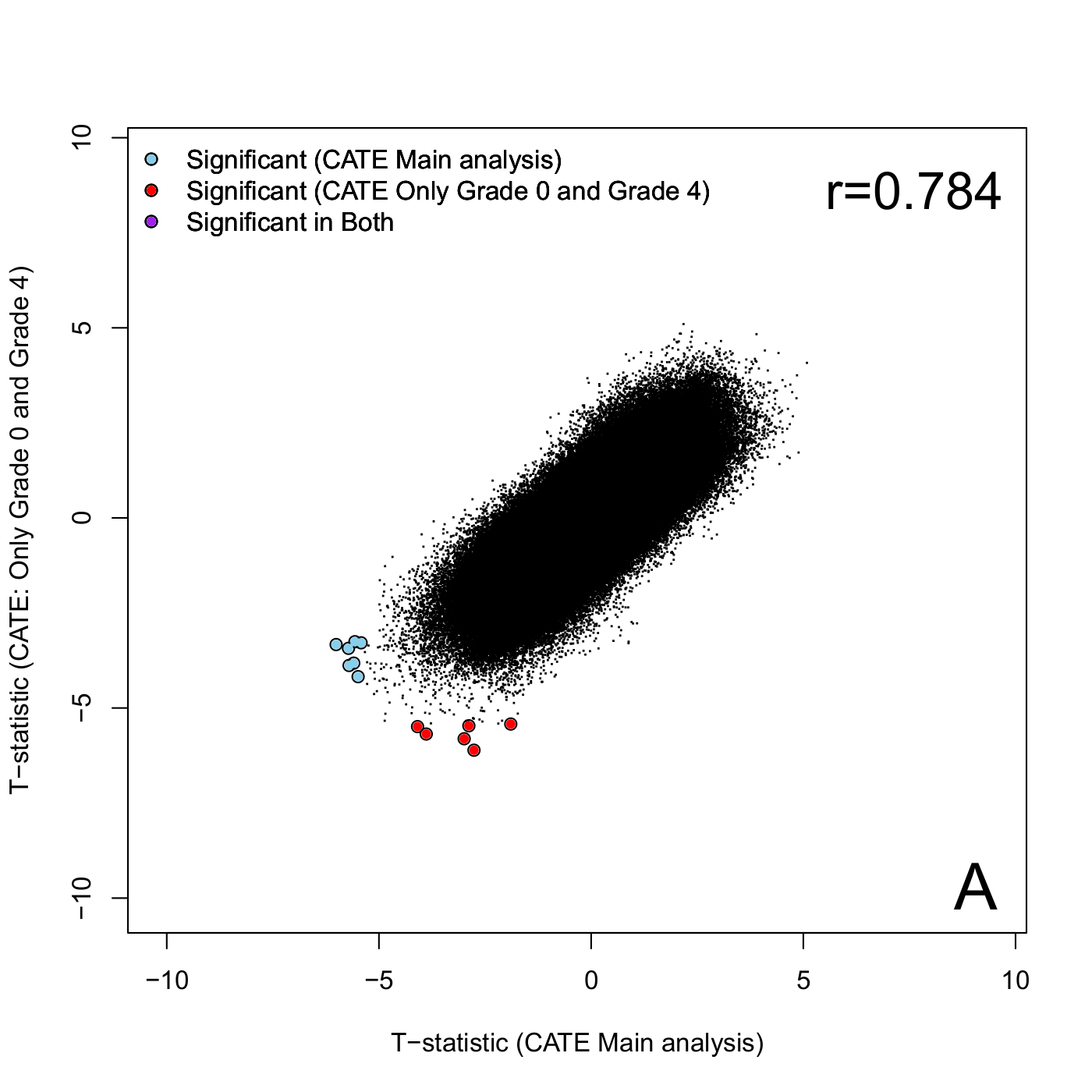

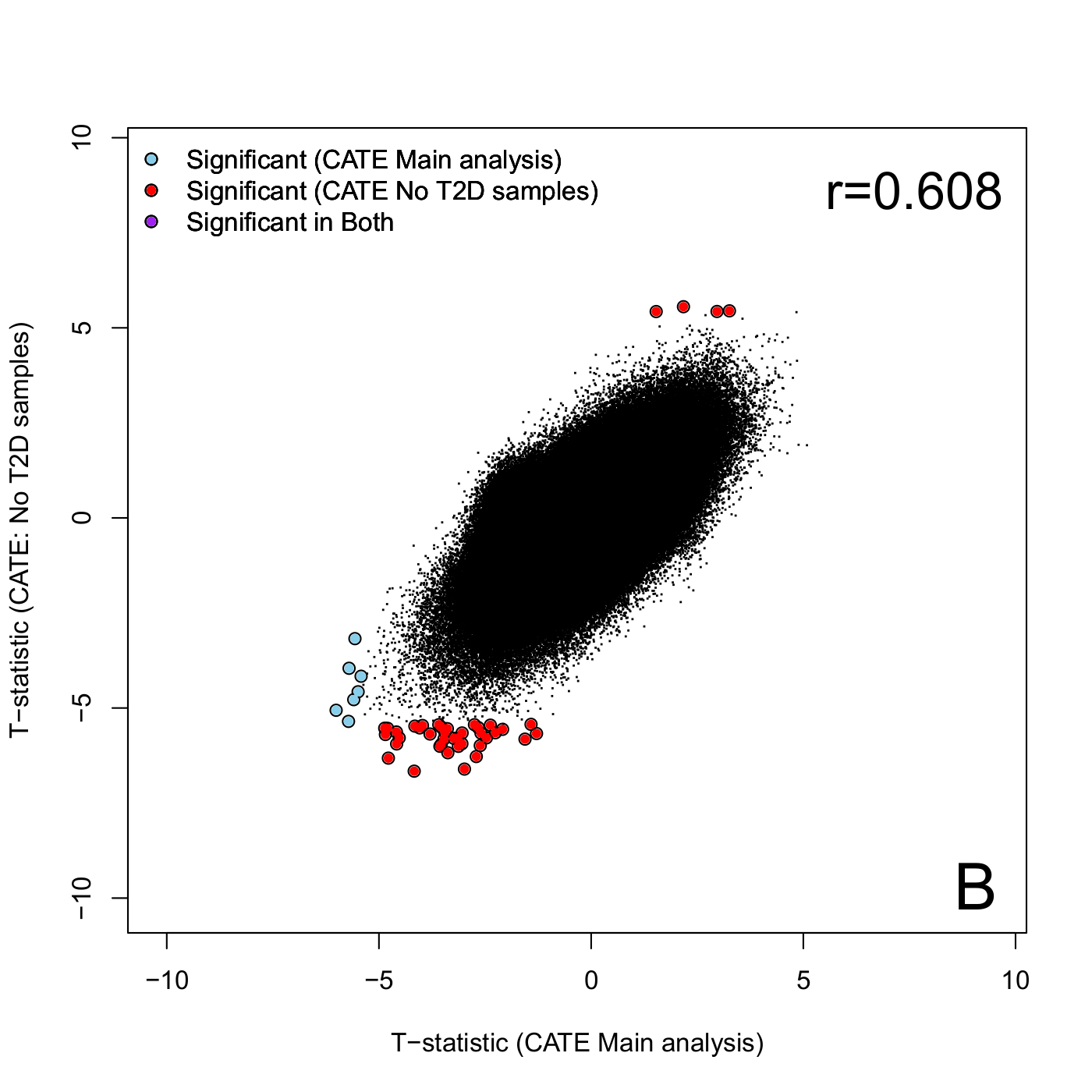

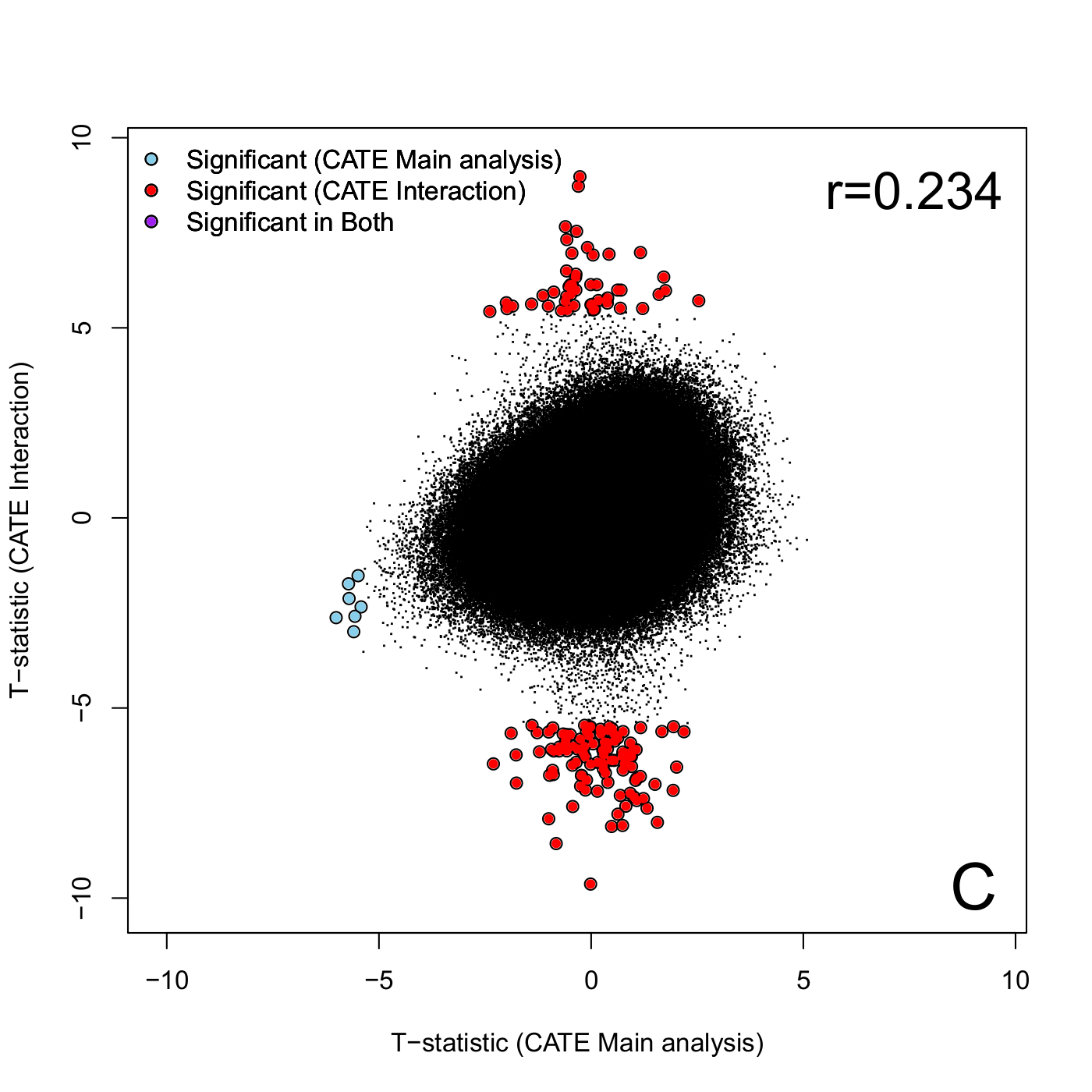

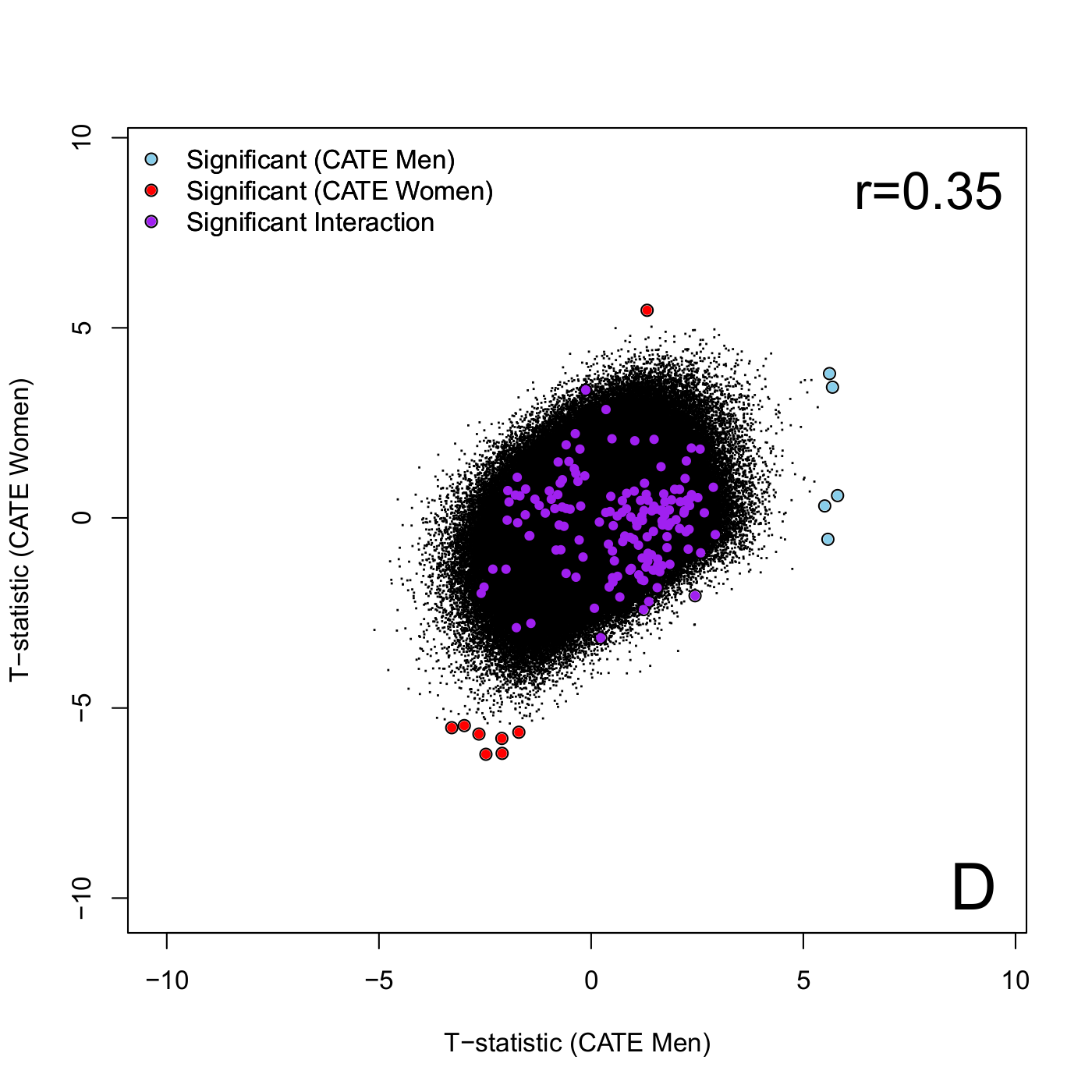


**Figure S6.** We performed sensitivity analyses to assess the robustness of the results including a comparison of the primary analysis to an analysis on individuals with extreme disease phenotypes (Grade 0 and Grade 4) (Panel A), a comparison of the primary analysis to non-T2D individuals (Panel B), a comparison of a sex*disease interaction analysis with the main analysis (Panel C), and a comparison of an analysis with only men to an analysis with only women (Panel D).


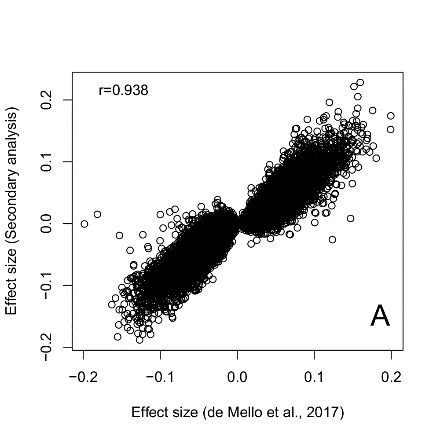

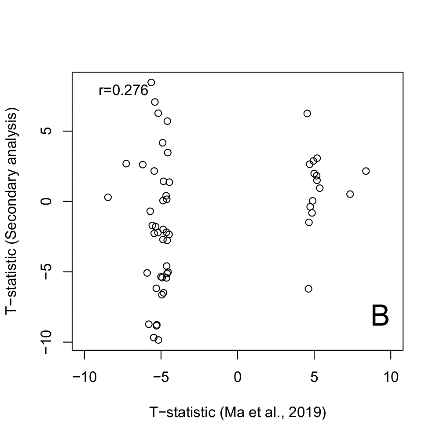

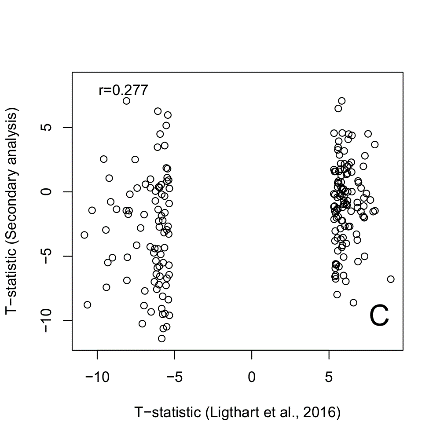


**Figure S7.** Comparison of our results with previous work. Effect sizes from our secondary analysis compared to a previous NASH-related EWAS in liver tissue (A). Test statistics from our secondary analysis compared to an EWAS of hepatic fat in blood (B), and an EWAS of inflammation (C-reactive protein) in blood (C).

**
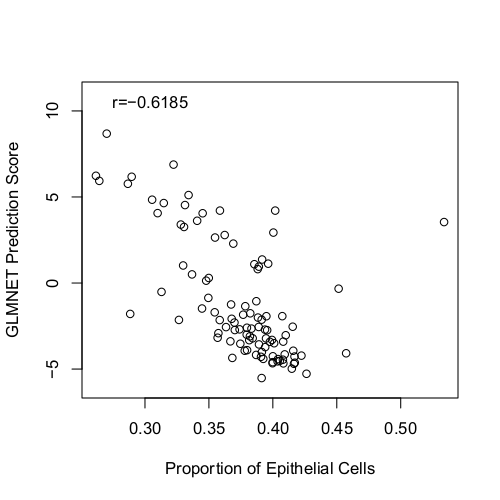

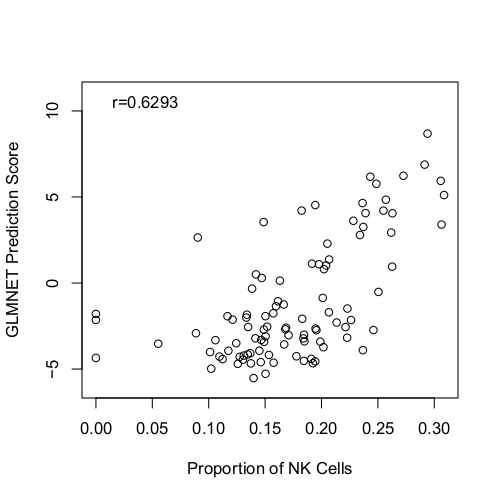

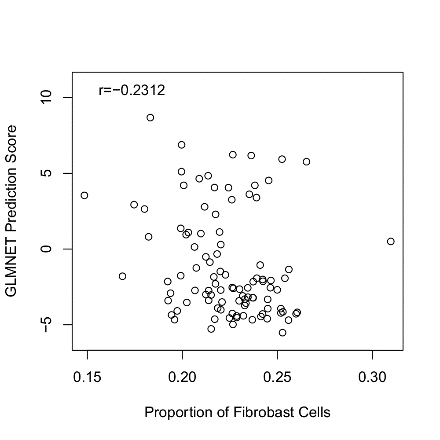

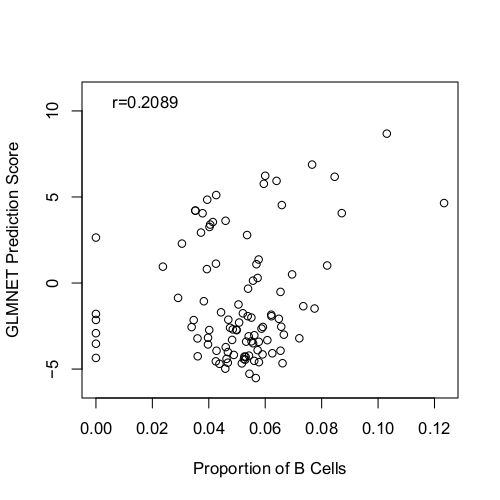

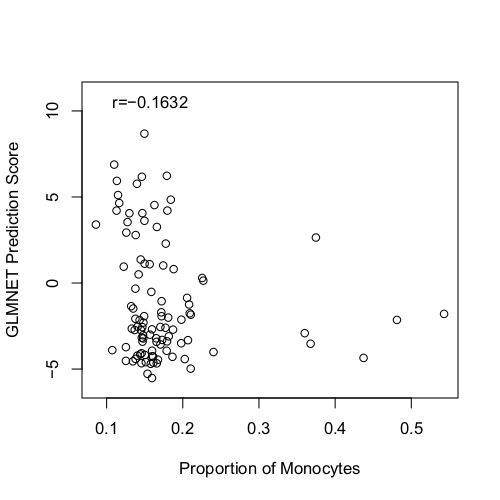
**

**Figure S8.** Elastic net prediction scores plotted against EpiDISH-inferred cell type proportions for epithelial cells, NK cells, fibroblast cells, B cells, and monocytes.

| **CpG** | **Chr** | **Position** | **Effect Size** | **T-statistic** | **p-value** | **Overlapping Gene** | **Enhancer ID** |
| --- | --- | --- | --- | --- | --- | --- | --- |
| cg01975877 | chr1 | 9264173 | 0.063 | 4.7 | 2.7E-06 | H6PD | GH01I009262 |
| cg17994196 | chr1 | 15290610 | -0.041 | -4.9 | 1.0E-06 | FHAD1 | GH01I015290 |
| cg05099985 | chr1 | 15919436 | -0.050 | -5.1 | 3.8E-07 | SPEN | GH01I015918 |
| cg16357353 | chr1 | 20411294 | -0.040 | -4.5 | 7.2E-06 | LINC01141 |  |
| cg14759787 | chr1 | 23673559 | -0.032 | -4.9 | 1.0E-06 | RP11-223J15.2 |  |
| cg12278018 | chr1 | 34800441 | -0.053 | -4.8 | 1.2E-06 | SMIM12 |  |
| cg17888086 | chr1 | 37479456 | -0.050 | -4.9 | 9.5E-07 | MIR6732;ZC3H12A |  |
| cg04497870 | chr1 | 41811613 | -0.034 | -4.5 | 5.7E-06 | HIVEP3 | GH01I041811 |
| cg05252126 | chr1 | 53413757 | -0.066 | -5.2 | 2.0E-07 | SLC25A3P1 | GH01I053411 |
| cg08133848 | chr1 | 84253995 | -0.110 | -4.9 | 9.5E-07 |  | GH01I084252 |
| cg20275771 | chr1 | 202012446 | -0.075 | -4.5 | 6.7E-06 | ELF3;RP11-510N19.5 |  |
| cg24000206 | chr1 | 218377420 | -0.111 | -5.4 | 8.0E-08 | TGFB2 | GH01I218373 |
| cg24686918 | chr1 | 221440316 | -0.102 | -4.5 | 6.6E-06 |  | GH01I221437 |
| cg06092869 | chr1 | 225455044 | -0.079 | -4.6 | 4.6E-06 | RP11-496N12.6 | GH01I225454 |
| cg15798997 | chr1 | 230851602 | -0.043 | -4.7 | 3.2E-06 | C1orf198 | GH01I230848 |
| cg04580625 | chr2 | 7825871 | -0.090 | -4.7 | 2.8E-06 |  | GH02I007824 |
| cg09936080 | chr2 | 43426646 | -0.047 | -4.5 | 6.0E-06 | THADA | GH02I043423 |
| cg25400574 | chr2 | 65577005 | -0.097 | -4.7 | 2.7E-06 | AC074391.1 | GH02I065576 |
| cg26166371 | chr2 | 87452979 | -0.074 | -4.8 | 1.5E-06 | AC133644.2 |  |
| cg05701495 | chr2 | 102031314 | 0.103 | 4.7 | 2.2E-06 |  | GH02I102030 |
| cg08035771 | chr2 | 111497547 | -0.076 | -4.7 | 3.2E-06 | AC017002.1;RP11-68E19.2 |  |
| cg10532358 | chr2 | 160224218 | -0.069 | -4.8 | 1.3E-06 | ITGB6 | GH02I160219 |
| cg18259342 | chr2 | 218320136 | 0.067 | 4.8 | 1.3E-06 | PNKD |  |
| cg23153680 | chr2 | 219219194 | 0.067 | 4.6 | 3.9E-06 | ABCB6;ATG9A |  |
| cg06716437 | chr2 | 219219229 | 0.048 | 4.5 | 5.9E-06 | ABCB6;ATG9A |  |
| cg09901892 | chr2 | 224023221 | -0.086 | -4.7 | 3.2E-06 | SERPINE2 |  |
| cg02273014 | chr2 | 226115220 | -0.101 | -5.1 | 3.5E-07 |  | GH02I226113 |
| cg08532185 | chr3 | 2950423 | -0.117 | -4.9 | 1.1E-06 | CNTN4 |  |
| cg21646392 | chr3 | 23668812 | -0.083 | -4.5 | 6.5E-06 |  | GH03I023667 |
| cg19538677 | chr3 | 27534235 | -0.070 | -4.9 | 9.2E-07 |  | GH03I027531 |
| cg04246167 | chr3 | 58998739 | -0.035 | -4.6 | 4.5E-06 | C3orf67;C3orf67-AS1 | GH03I058996 |
| cg18560366 | chr3 | 109338717 | 0.076 | 4.5 | 6.5E-06 | DPPA4 |  |
| cg21756208 | chr3 | 134427563 | -0.060 | -4.8 | 1.8E-06 |  | GH03I134426 |
| cg23460823 | chr3 | 170726846 | -0.043 | -4.7 | 2.7E-06 | CLDN11 | GH03I170723 |
| cg26300517 | chr4 | 38731040 | -0.040 | -4.5 | 6.8E-06 |  | GH04I038730 |
| cg07645190 | chr5 | 56211561 | -0.109 | -4.9 | 1.1E-06 | ANKRD55 | GH05I056210 |
| cg10282890 | chr5 | 74316057 | -0.088 | -5.0 | 7.4E-07 |  |  |
| cg18360579 | chr5 | 113489569 | -0.068 | -4.8 | 1.4E-06 | MCC |  |
| cg20708282 | chr5 | 127898991 | -0.039 | -4.5 | 6.3E-06 | LINC01183 |  |
| cg19607021 | chr5 | 139725248 | -0.067 | -4.6 | 3.7E-06 | CTB-35F21.1 | GH05I139724 |
| cg21430814 | chr5 | 147906512 | -0.095 | -5.0 | 6.3E-07 | C5orf46 |  |
| cg23336160 | chr5 | 148969365 | -0.039 | -4.7 | 2.0E-06 | RP11-44B19.1;SH3TC2 | GH05I148964 |
| cg25020590 | chr5 | 157602797 | -0.039 | -4.6 | 4.4E-06 |  | GH05I157602 |
| cg11843868 | chr5 | 158382705 | -0.092 | -4.8 | 1.5E-06 | RP11-524N5.1 | GH05I158381 |
| cg19979108 | chr5 | 172443871 | -0.101 | -4.9 | 1.1E-06 | SH3PXD2B | GH05I172442 |
| cg08119046 | chr6 | 14542875 | -0.044 | -4.7 | 2.4E-06 |  | GH06I014542 |
| cg13027088 | chr6 | 53929030 | -0.130 | -5.0 | 6.0E-07 | MLIP;RP11-411K7.1 | GH06I053926 |
| cg09822959 | chr6 | 82862789 | -0.159 | -5.4 | 5.9E-08 |  |  |
| cg13306815 | chr6 | 140043474 | -0.057 | -4.5 | 7.4E-06 |  | GH06I140042 |
| cg25784969 | chr6 | 144264560 | -0.090 | -4.5 | 6.2E-06 |  | GH06I144263 |
| cg06623218 | chr7 | 27237960 | -0.034 | -4.6 | 5.3E-06 |  |  |
| cg24260606 | chr7 | 39428451 | -0.081 | -4.5 | 5.8E-06 | POU6F2 |  |
| cg23624444 | chr7 | 41108211 | -0.097 | -4.6 | 4.0E-06 | LINC01449 | GH07I041106 |
| cg02426774 | chr7 | 50869532 | -0.129 | -4.6 | 3.5E-06 | RP4-724E13.2 |  |
| cg05127193 | chr7 | 106425540 | -0.047 | -4.7 | 3.1E-06 | CTB-111H14.1 | GH07I106423 |
| cg15403056 | chr7 | 117435735 | -0.157 | -4.9 | 1.0E-06 |  |  |
| cg15928106 | chr7 | 130961319 | -0.047 | -4.6 | 3.6E-06 | LINC-PINT | GH07I130954 |
| cg19686543 | chr7 | 148327678 | -0.093 | -6.0 | 1.9E-09 | CNTNAP2 |  |
| cg07999732 | chr7 | 150383754 | 0.096 | 4.6 | 3.5E-06 | RP4-584D14.6;ZNF775 |  |
| cg14628604 | chr7 | 157854009 | -0.049 | -5.2 | 2.0E-07 | AC011899.9;PTPRN2 | GH07I157853 |
| cg24921221 | chr8 | 12726683 | -0.106 | -4.7 | 2.3E-06 | LONRF1 | GH08I012725 |
| cg27527262 | chr8 | 79869515 | -0.099 | -5.2 | 1.6E-07 | RP11-26J3.1 |  |
| cg20788020 | chr8 | 104665273 | -0.073 | -4.7 | 3.3E-06 | ZFPM2 | GH08I104664 |
| cg19839406 | chr8 | 135580236 | -0.061 | -4.5 | 7.2E-06 | KHDRBS3 | GH08I135579 |
| cg15089921 | chr9 | 3962860 | -0.126 | -4.9 | 1.1E-06 | GLIS3 | GH09I003962 |
| cg11227425 | chr9 | 37954152 | -0.062 | -5.0 | 6.4E-07 | RP11-613M10.9;SHB | GH09I037949 |
| cg19554338 | chr9 | 86792317 | -0.066 | -4.8 | 1.7E-06 |  |  |
| cg08033828 | chr9 | 89137984 | -0.093 | -5.6 | 2.6E-08 | SHC3 | GH09I089136 |
| cg07796897 | chr10 | 5976262 | -0.115 | -4.6 | 4.6E-06 | IL15RA |  |
| cg08382732 | chr10 | 19660051 | -0.061 | -4.9 | 1.0E-06 | MALRD1 |  |
| cg18941917 | chr10 | 33134214 | -0.096 | -5.3 | 9.8E-08 |  | GH10I033133 |
| cg20661965 | chr10 | 46375779 | -0.055 | -4.6 | 3.5E-06 | ANXA8L1;CH17-335B8.4 |  |
| cg23572703 | chr10 | 60481433 | -0.121 | -4.5 | 7.0E-06 | ANK3 | GH10I060480 |
| cg14701867 | chr10 | 62433309 | -0.104 | -4.5 | 6.1E-06 | ZNF365 | GH10I062432 |
| cg05550145 | chr10 | 71871455 | -0.174 | -5.7 | 1.1E-08 |  | GH10I071870 |
| cg09531361 | chr10 | 72676575 | -0.065 | -5.3 | 1.3E-07 |  | GH10I072675 |
| cg09998038 | chr10 | 73894805 | -0.052 | -5.5 | 4.0E-08 |  | GH10I073886 |
| cg26568031 | chr10 | 89553623 | -0.084 | -5.0 | 4.4E-07 | SLC16A12 | GH10I089552 |
| cg03210827 | chr10 | 102815149 | 0.102 | 4.7 | 2.9E-06 | WBP1L | GH10I102811 |
| cg09417937 | chr11 | 1277688 | 0.050 | 5.1 | 3.7E-07 | TOLLIP | GH11I001274 |
| cg08750946 | chr11 | 14337301 | -0.054 | -4.8 | 1.6E-06 | RRAS2 |  |
| cg19323289 | chr11 | 19442356 | -0.059 | -4.5 | 6.4E-06 | NAV2 | GH11I019441 |
| cg02768790 | chr11 | 69337619 | -0.072 | -5.0 | 5.1E-07 | MYEOV | GH11I069336 |
| cg01926616 | chr11 | 115925349 | -0.070 | -5.1 | 3.1E-07 |  |  |
| cg06545916 | chr11 | 127274572 | -0.075 | -4.5 | 6.5E-06 | RP11-480C22.1 |  |
| cg12301841 | chr12 | 18383514 | -0.129 | -4.6 | 4.8E-06 | PIK3C2G |  |
| cg16663952 | chr12 | 24199345 | -0.043 | -4.5 | 6.2E-06 |  |  |
| cg21242123 | chr12 | 52244350 | -0.069 | -4.8 | 1.3E-06 | KRT7 | GH12I052242 |
| cg24813323 | chr12 | 62848747 | -0.060 | -5.0 | 6.2E-07 | PPM1H |  |
| cg11404915 | chr12 | 127125926 | -0.110 | -5.0 | 6.9E-07 |  | GH12I127125 |
| cg08595439 | chr13 | 42405891 | -0.092 | -4.5 | 5.4E-06 |  | GH13I042405 |
| cg07461296 | chr13 | 108957767 | -0.141 | -4.8 | 1.7E-06 | MYO16 | GH13I108956 |
| cg00880833 | chr14 | 54107141 | -0.129 | -4.5 | 5.9E-06 |  | GH14I054104 |
| cg10534873 | chr14 | 56125184 | -0.063 | -4.9 | 1.2E-06 | PELI2 |  |
| cg18364371 | chr14 | 56352296 | -0.051 | -4.7 | 3.2E-06 |  |  |
| cg00807828 | chr14 | 96267499 | -0.065 | -4.8 | 1.6E-06 | BDKRB1;RP11-404P21.3 | GH14I096264 |
| cg18039797 | chr15 | 40217317 | 0.089 | 4.9 | 1.2E-06 | BUB1B;PAK6 |  |
| cg09031828 | chr15 | 59339649 | -0.040 | -4.5 | 7.2E-06 | MYO1E | GH15I059338 |
| cg09870609 | chr15 | 81410611 | -0.098 | -4.6 | 3.5E-06 | CTD-2240J17.1 |  |
| cg03547319 | chr16 | 4517888 | 0.060 | 4.5 | 6.8E-06 | CDIP1 |  |
| cg05009047 | chr16 | 4609465 | 0.104 | 4.8 | 2.0E-06 | UBALD1 |  |
| cg03815480 | chr16 | 48458528 | -0.052 | -4.8 | 1.5E-06 |  | GH16I048457 |
| cg04458219 | chr16 | 70702129 | -0.056 | -4.7 | 2.9E-06 | VAC14 |  |
| cg02660643 | chr16 | 87457981 | -0.045 | -4.9 | 1.1E-06 | ZCCHC14 | GH16I087455 |
| cg12439163 | chr17 | 5262508 | -0.065 | -4.5 | 5.6E-06 |  |  |
| cg17897629 | chr17 | 9389420 | -0.129 | -4.6 | 5.1E-06 | STX8 | GH17I009388 |
| cg23875758 | chr17 | 17812206 | 0.037 | 4.6 | 4.7E-06 | SREBF1 | GH17I017808 |
| cg02092885 | chr17 | 31052440 | -0.056 | -4.8 | 1.5E-06 | RP11-271K11.5;RP11-848P1.9 | GH17I031051 |
| cg22317887 | chr17 | 37698375 | -0.067 | -5.7 | 1.2E-08 | HNF1B |  |
| cg24393783 | chr17 | 72127024 | -0.076 | -4.5 | 6.0E-06 | SOX9-AS1 |  |
| cg05669282 | chr17 | 72137029 | -0.128 | -5.0 | 6.4E-07 | SOX9-AS1 | GH17I072134 |
| cg22384905 | chr17 | 72702552 | -0.103 | -5.3 | 1.3E-07 | SLC39A11 | GH17I072702 |
| cg18733548 | chr17 | 79793018 | 0.064 | 4.5 | 6.5E-06 | CBX8 | GH17I079792 |
| cg16548344 | chr18 | 26460896 | -0.079 | -4.7 | 2.1E-06 | KCTD1 | GH18I026460 |
| cg25000618 | chr18 | 36786913 | -0.104 | -4.7 | 2.5E-06 | TPGS2 | GH18I036786 |
| cg05762640 | chr19 | 2616887 | 0.085 | 4.5 | 6.5E-06 | GNG7 | GH19I002615 |
| cg23004527 | chr19 | 3390355 | 0.062 | 4.7 | 2.8E-06 | NFIC | GH19I003386 |
| cg18317135 | chr19 | 4677218 | 0.057 | 4.6 | 3.8E-06 | DPP9 | GH19I004676 |
| cg07230018 | chr19 | 4677270 | 0.065 | 4.9 | 1.0E-06 | DPP9 | GH19I004676 |
| cg16303737 | chr20 | 553651 | -0.105 | -4.8 | 1.8E-06 |  |  |
| cg02396633 | chr20 | 10556251 | -0.129 | -4.7 | 2.7E-06 | SLX4IP | GH20I010555 |
| cg16582186 | chr20 | 10954602 | -0.083 | -4.6 | 3.7E-06 | RP11-103J8.1 |  |
| cg11793257 | chr20 | 25238083 | -0.072 | -5.1 | 3.2E-07 |  | GH20I025232 |
| cg01078772 | chr20 | 32332683 | -0.079 | -4.6 | 3.9E-06 | KIF3B | GH20I032331 |
| cg01931861 | chr21 | 41601636 | -0.077 | -5.6 | 2.2E-08 |  | GH21I041601 |
| cg10747242 | chr21 | 43613923 | -0.049 | -4.6 | 5.1E-06 | HSF2BP | GH21I043612 |
| cg11509179 | chr22 | 30207156 | -0.087 | -4.7 | 2.4E-06 | RP3-438O4.4 | GH22I030201 |
| cg08933688 | chr22 | 30207329 | -0.092 | -4.9 | 7.8E-07 | RP3-438O4.4 | GH22I030201 |

**Table S1.** Summary information of the 128 fibrosis-related CpGs that passed less stringent threshold (FDR<0.05)

| **ONTOLOGY** | **TERM** | **N** | **DE** | **P.DE** | **FDR** |
| --- | --- | --- | --- | --- | --- |
| CC | cytoplasm | 11436 | 6858 | 3.85E-26 | 8.76E-22 |
| BP | small molecule metabolic process | 1982 | 1315 | 1.62E-24 | 1.85E-20 |
| CC | extracellular exosome | 2115 | 1391 | 1.49E-23 | 1.13E-19 |
| CC | vesicle | 3999 | 2507 | 6.70E-23 | 3.81E-19 |
| BP | intracellular signal transduction | 2822 | 1866 | 9.26E-23 | 4.22E-19 |
| BP | regulation of signaling | 3647 | 2369 | 5.26E-22 | 2.00E-18 |
| BP | localization | 6693 | 4129 | 1.19E-21 | 3.63E-18 |
| BP | regulation of cell communication | 3607 | 2341 | 1.28E-21 | 3.63E-18 |
| BP | cell adhesion | 1466 | 1025 | 6.68E-21 | 1.65E-17 |
| BP | biological adhesion | 1473 | 1029 | 7.80E-21 | 1.65E-17 |

**Table S2.** Top 10 GO terms for the secondary analysis.

| **ENS ID** | **Gene Name** | **Position** | **cg01931861** | **cg05550145** | **cg08033828** | **cg09822959** | **cg09998038** | **cg19686543** | **cg22317887** |
| --- | --- | --- | --- | --- | --- | --- | --- | --- | --- |
| ENSG00000108448 | TRIM16L | chr17:18697998-18736118 | 4.8 | 5.1 |  | 5.1 |  | 5.1 |  |
| ENSG00000113597 | TRAPPC13 | chr5:65624716-65666233 | -5.2 |  | -5.1 | -5.1 |  | -5.1 |  |
| ENSG00000114209 | PDCD10 | chr3:167683298-167734939 | -5.0 |  | -5.8 | -5.4 |  | -5.3 |  |
| ENSG00000152926 | ZNF117 | chr7:64971776-65006684 | -6.3 |  |  | -4.7 |  | -5.9 | -5.3 |
| ENSG00000174992 | ZG16 | chr16:29778240-29782973 | 5.7 | 4.8 |  |  | 6.3 | 6.2 |  |
| ENSG00000204588 | LINC01123 | chr2:109987063-109996140 |  |  | 4.7 | 6.1 |  | 4.7 | 4.8 |
| ENSG00000205277 | MUC12 | chr7:100969623-101018949 | 5.4 | 6.8 |  | 6.6 |  | 5.6 |  |
| ENSG00000215156 | RP11-1023L17.2 | chr5:34190056-34193653 | 4.7 | 5.5 |  | 6.2 |  |  | 5.5 |
| ENSG00000276710 | CSPG4P10 | chr15:82459472-82477258 | 4.8 | 5.4 | 5.5 | 5.9 |  |  |  |
| ENSG00000120053 | GOT1 | chr10:99396870-99430624 | 5.7 | 4.7 |  |  | 4.7 | 5.9 | 5.0 |
| ENSG00000161692 | DBF4B | chr17:44708608-44752264 | 4.8 | 5.6 | 4.9 | 6.2 |  |  | 4.9 |
| ENSG00000223839 | FAM95B1 | chr9:40321299-40329221 | 4.8 | 5.0 |  | 5.5 | 5.1 |  | 4.9 |
| ENSG00000230338 | MTND4P19 | chr10:94774156-94774633 | 4.9 | 4.9 | 5.3 | 5.0 |  | 5.4 |  |
| ENSG00000272398 | CD24 | chr6:106969831-106975627 | -5.1 | -5.0 | -5.0 |  | -5.2 | -5.3 |  |
| ENSG00000274628 | FAM95B1 | chr9:66047084-66055006 | 4.9 | 5.3 |  | 5.9 | 5.3 |  | 5.2 |
| ENSG00000135679 | MDM2 | chr12:68808176-68850686 | -5.0 | -5.3 | -5.3 | -5.5 |  | -5.0 | -4.7 |
| ENSG00000137101 | CD72 | chr9:35609533-35646810 | 4.9 | 4.9 | 5.5 | 5.4 |  | 5.2 | 5.0 |
| ENSG00000149573 | MPZL2 | chr11:118253403-118264536 | -5.8 | -5.9 | -5.3 | -5.3 |  | -5.1 | -4.7 |
| ENSG00000164199 | ADGRV1 | chr5:90529344-91164221 | 5.9 | 6.1 |  | 6.0 | 5.4 | 6.1 | 6.0 |
| ENSG00000172667 | ZMAT3 | chr3:179017223-179072279 | -6.2 | -5.5 | -6.3 | -4.8 |  | -7.4 | -5.4 |
| ENSG00000186648 | CARMIL3 | chr14:24052000-24069728 | 5.2 | 5.9 | 4.9 | 4.8 | 4.7 |  | 5.0 |
| ENSG00000196090 | PTPRT | chr20:42072752-43189970 | 6.0 | 5.8 | 6.0 | 6.0 |  | 6.0 | 7.0 |
| ENSG00000278996 | CH507-513H4.1 | chr21:8197620-8227646 | 5.5 | 6.7 | 5.4 | 7.2 |  | 5.1 | 5.6 |
| ENSG00000280441 | CH507-528H12.1 | chr21:8380665-8410645 | 5.5 | 6.6 | 5.4 | 7.2 |  | 5.1 | 5.6 |

**Table S3.** Association between expression of genes and DNAm of CpGs for genes with significant associations at ≥4 CpGs. Columns 4-10 indicate the test statistic corresponding to the association between the CpG in the column with the expression at the gene in the row.

| **CpG** | **Cis: within gene or <50 kb upstream** | **Distal (same chr., ≥50 kb)** | **Trans (different chr.)** |
| --- | --- | --- | --- |
| cg09822959 | 0 | 3 | 199 |
| cg19686543 | 0 | 4 | 48 |
| cg08033828 | 0 | 2 | 29 |
| cg05550145 | 0 | 5 | 78 |
| cg09998038 | 0 | 2 | 12 |
| cg22317887 | 0 | 2 | 33 |
| cg01931861 | 0 | 3 | 51 |

**Table S4.** Location of genes relative to the CpG.

| **cg09822959** | **cg09998038** |
| --- | --- |
| cell projection morphogenesis, cell morphogenesis involved in differentiation, homophilic cell adhesion via plasma membrane adhesion molecules, cellular component morphogenesis, cellular response to caffeine, photoreceptor cell maintenance, mitotic chromosome movement towards spindle pole | regulation of lung blood pressure, response to transition metal nanoparticle, tricuspid valve morphogenesis, negative regulation of cell proliferation involved in heart valve morphogenesis, aspartate biosynthetic process, glutamate catabolic process to aspartate, glutamate catabolic process to 2-oxoglutarate, negative regulation of chondrocyte proliferation, cell proliferation involved in heart valve development, regulation of transmembrane receptor protein serine/threonine kinase signaling pathway, glycerol biosynthetic process, negative regulation of mitochondrial depolarization, transdifferentiation, lymphatic endothelial cell differentiation, aspartate catabolic process, negative regulation of vasoconstriction, pharyngeal arch artery morphogenesis, negative regulation of collagen biosynthetic process, positive regulation of axon extension involved in axon guidance, negative regulation of cytosolic calcium ion concentration, regulation of neurotransmitter receptor localization to postsynaptic specialization membrane, negative regulation of systemic arterial blood pressure, mitral valve morphogenesis, oxaloacetate metabolic process, atrial septum morphogenesis, regulation of cell proliferation involved in heart morphogenesis, lymphangiogenesis, fatty acid homeostasis, retina vasculature development in camera-type eye, response to immobilization stress, protein localization to postsynaptic specialization membrane, venous blood vessel development, regulation of ARF protein signal transduction, regulation of protein localization to synapse, pulmonary valve development, chondrocyte development, outflow tract septum morphogenesis, positive regulation of cartilage development, atrioventricular valve development, cardiac atrium development, glutamine family amino acid catabolic process, negative regulation of animal organ morphogenesis, alditol metabolic process, positive regulation of transforming growth factor beta receptor signaling pathway, maternal placenta development, aortic valve development, positive regulation of pathway-restricted SMAD protein phosphorylation, positive regulation of bone mineralization, positive regulation of BMP signaling pathway, endocardial cushion development, regulation of collagen metabolic process, ventricular septum morphogenesis, positive regulation of biomineralization, neuron projection extension involved in neuron projection guidance, regulation of membrane depolarization, glutamate metabolic process, activin receptor signaling pathway |

**Table S5.** Significant GO Terms (FDR<0.05) for the set of genes whose expression significantly associated with DNAm at 2 of the 7 fibrosis-related CpG sites. cg09822959 had 7 significant terms and cg09998038 had 57 significant terms. The other 5 CpGs had no significant GO Terms.

| **GO Term** | **P-value** | **FDR** |
| --- | --- | --- |
| aspartate biosynthetic process | 1.36E-03 | 0.0583 |
| glutamate catabolic process to aspartate | 1.36E-03 | 0.0583 |
| glutamate catabolic process to 2-oxoglutarate | 1.36E-03 | 0.0583 |
| B cell receptor transport into membrane raft | 1.36E-03 | 0.0583 |
| chemokine receptor transport out of membrane raft | 1.36E-03 | 0.0583 |
| negative regulation of transforming growth factor beta3 production | 1.36E-03 | 0.0583 |
| transcription factor catabolic process | 1.36E-03 | 0.0583 |
| cellular response to vitamin B1 | 1.36E-03 | 0.0583 |
| response to formaldehyde | 1.36E-03 | 0.0583 |
| response to transition metal nanoparticle | 1.36E-03 | 0.0583 |
| aspartate catabolic process | 2.72E-03 | 0.0647 |
| intrinsic apoptotic signaling pathway in response to hydrogen peroxide | 2.72E-03 | 0.0647 |
| transdifferentiation | 2.72E-03 | 0.0647 |
| glomerular parietal epithelial cell differentiation | 2.72E-03 | 0.0647 |
| cellular response to actinomycin D | 2.72E-03 | 0.0647 |
| Golgi reassembly | 2.72E-03 | 0.0647 |
| response to water-immersion restraint stress | 2.72E-03 | 0.0647 |
| positive regulation of nephron tubule epithelial cell differentiation | 2.72E-03 | 0.0647 |
| intrinsic apoptotic signaling pathway | 2.89E-03 | 0.0653 |
| homophilic cell adhesion via plasma membrane adhesion molecules | 3.43E-03 | 0.0736 |
| glycerol biosynthetic process | 4.07E-03 | 0.0751 |
| traversing start control point of mitotic cell cycle | 4.07E-03 | 0.0751 |
| peptidyl-tyrosine dephosphorylation involved in inactivation of protein kinase activity | 4.07E-03 | 0.0751 |
| oxaloacetate metabolic process | 5.42E-03 | 0.0751 |
| protein transport within lipid bilayer | 5.42E-03 | 0.0751 |
| negative regulation of collagen biosynthetic process | 5.42E-03 | 0.0751 |
| establishment of Golgi localization | 5.42E-03 | 0.0751 |
| negative regulation of mitochondrial depolarization | 5.42E-03 | 0.0751 |
| cellular response to UV-C | 5.42E-03 | 0.0751 |
| protein localization to membrane raft | 5.42E-03 | 0.0751 |
| negative regulation of blood vessel endothelial cell proliferation involved in sprouting angiogenesis | 5.42E-03 | 0.0751 |
| response to inorganic substance | 6.36E-03 | 0.0852 |
| response to ether | 6.78E-03 | 0.0881 |
| negative regulation of cytosolic calcium ion concentration | 8.13E-03 | 0.0996 |
| programmed cell death in response to reactive oxygen species | 8.13E-03 | 0.0996 |

**Table S6.** Top GO terms of the genes with 4 or more NAFLD-related CpGs associated with gene expression.

| **Model coefficients** | |
| --- | --- |
| (Intercept) | 34.62704503 |
| cg05240210 | -0.1990985933 |
| cg08262559 | -2.212637279 |
| cg18259342 | 2.024037569 |
| cg14006804 | -2.470660916 |
| cg22802793 | -3.887815084 |
| cg25784969 | -0.6117759119 |
| cg14628604 | -2.808585383 |
| cg01136183 | -11.22682875 |
| cg20788020 | -1.886838261 |
| cg21789008 | -2.92978426 |
| cg21208274 | -5.434679532 |
| cg15180531 | -0.4287537858 |
| cg16809873 | -1.748701366 |
| cg23433822 | -1.008720724 |
| cg19573117 | 2.013753649 |
| cg00689534 | -0.3335840201 |
| cg25104066 | 5.929970675 |
| cg08160460 | 2.996895244 |
| cg04862556 | 0.9534805309 |
| cg21082028 | 0.4205837729 |
| cg09283991 | -7.763270428 |
| cg13270625 | -5.009892741 |
| cg23004527 | 3.966035253 |
| cg02396633 | -0.3640014019 |
| cg21538115 | -0.1196883885 |
| cg21751556 | -0.1931580158 |
| cg00705130 | -6.711220393 |

**Table S7.** Elastic net coefficients for each CpG site.
